# COVID-19 alters human microbiomes: a meta-analysis

**DOI:** 10.3389/fcimb.2023.1211348

**Published:** 2023-08-02

**Authors:** Rine Christopher Reuben, Rémy Beugnon, Stephanie D. Jurburg

**Affiliations:** ^1^ German Centre of Integrative Biodiversity Research (iDiv) Halle-Jena-Leipzig, Leipzig, Germany; ^2^ Institute of Biology, Leipzig University, Leipzig, Germany; ^3^ Leipzig Institute for Meteorology, Universität Leipzig, Leipzig, Germany; ^4^ CEFE, Université de Montpellier, CNRS, EPHE, IRD, Montpellier, France; ^5^ Department of Environmental Microbiology, Helmholtz Centre for Environmental Research - UFZ, Leipzig, Germany

**Keywords:** COVID-19, gut microbiome, host health, SARS-C0V-2, infection, human microbiome

## Abstract

**Introduction:**

Severe acute respiratory syndrome coronavirus-2 (SARS-CoV-2) has infected a substantial portion of the world’s population, and novel consequences of COVID-19 on the human body are continuously being uncovered. The human microbiome plays an essential role in host health and well-being, and multiple studies targeting specific populations have reported altered microbiomes in patients infected with SARS-CoV-2. Given the global scale and massive incidence of COVID on the global population, determining whether the effects of COVID-19 on the human microbiome are consistent and generalizable across populations is essential.

**Methods:**

We performed a synthesis of human microbiome responses to COVID-19. We collected 16S rRNA gene amplicon sequence data from 11 studies sampling the oral and nasopharyngeal or gut microbiome of COVID-19-infected and uninfected subjects. Our synthesis included 1,159 respiratory (oral and nasopharyngeal) microbiome samples and 267 gut microbiome samples from patients in 11 cities across four countries.

**Results:**

Our reanalyses revealed communitywide alterations in the respiratory and gut microbiomes across human populations. We found significant overall reductions in the gut microbial diversity of COVID-19-infected patients, but not in the respiratory microbiome. Furthermore, we found more consistent community shifts in the gut microbiomes of infected patients than in the respiratory microbiomes, although the microbiomes in both sites exhibited higher host-to-host variation in infected patients. In respiratory microbiomes, COVID-19 infection resulted in an increase in the relative abundance of potentially pathogenic bacteria, including *Mycoplasma*.

**Discussion:**

Our findings shed light on the impact of COVID-19 on the human-associated microbiome across populations, and highlight the need for further research into the relationship between long-term effects of COVID-19 and altered microbiota.

## Introduction

The emergence and rapid spread of the novel beta-coronavirus, severe acute respiratory syndrome coronavirus-2 (SARS-CoV-2), caused severe and unprecedented public health and socioeconomic challenges globally (Waters et al., 2022). Primarily, COVID-19 presents as a multifaceted and multi-organ infection with variable severity. Symptoms range from acute respiratory distress syndrome to pneumonia and include non-specific flu-like symptoms, gastrointestinal symptoms, myocardial dysfunction, multiple organ failure, and death (Kumar Singh et al., 2019; Baud et al., 2020; Onder et al., 2020). In most cases, SARS-CoV-2-infected persons are either asymptomatic or show mild symptoms. However, approximately 5% of those infected, usually the elderly and/or individuals with comorbidities, develop a severe form of the disease, resulting in intensive medical care and death (Grasselli et al., 2020; Wiersinga et al., 2020). As of April 2023, there have been 762,791,152 confirmed COVID-19 cases with 6,897,025 mortalities worldwide (WHO Coronavirus (COVID-19) Dashboard).

Among the many long-term effects associated with COVID-19 infection, numerous studies have reported altered microbiota in COVID-19 patients. The human microbiome plays a vital role in host health (Kumpitsch et al., 2019) and has been suggested to act as an additional organ (Baquero and Nombela, 2012). These microbial communities maintain host homeostasis through complex and essential interactions, which result in improved immunomodulation, metabolism, organ functions, mucosal barrier integrity, and structural protection against intruding pathogens (Jandhyala et al., 2015; Kumpitsch et al., 2019). Specific microbial communities are associated with different human tissues (Human Microbiome Project Consortium, 2012; Pflughoeft and Versalovic, 2012).

Perturbations such as COVID-19 can result in microbiome dysbiosis in human microbiomes, in which the composition and diversity of beneficial and/or commensal microorganisms are altered, promoting the growth or opportunistic pathogens (Hoque et al., 2021b; Ren et al., 2021; Sun et al., 2022). In particular, the observation of increased host-to-host variability in the microbiomes associated with unhealthy hosts has been dubbed the Anna Karenina Principle (AKP), derived from the opening line of Tolstoy’s *Anna Karenina*: “All happy families are all alike; each unhappy family is unhappy in its own way”.

Different human diseases including obesity, psoriasis, arthritis, inflammatory bowel disease (IBD), influenza, HBV, and HIV have been reported to significantly alter human microbiomes (Ling et al., 2015; Lu et al., 2017; Gilbert et al., 2018; Gonçalves et al., 2019; Yun et al., 2019; Sencio et al., 2021). Similarly, several reports have demonstrated changes in the microbiomes (intestinal, nasopharyngeal, and oral) of COVID-19 patients during active infection and convalescent state, and these are usually characterized by the depletion of beneficial commensal microbes and a higher abundance of opportunistic pathogens (Zuo et al., 2020; Zuo et al., 2020; Hoque et al., 2021a; Hoque et al., 2021b; Jochems et al., 2021; Ren et al., 2021; Xu et al., 2021; Yeoh et al., 2021). The composition and diversity of the gut, nasal, or oral microbiome of COVID-19 patients are now widely believed to be predictive of COVID-19 prognosis, progression, and severity (Mathieu et al., 2018; Wypych et al., 2019; Chen et al., 2020; He et al., 2020; Hoque et al., 2021b). Moreover, the abundance of specific microbes within human microbiomes are now identified as biomarkers to distinguish COVID-19-infected individuals from healthy persons (Gou et al., 2020; Zuo et al., 2020; Tian et al., 2021).

Extensive interactions exist between the host immune system and microbiome. These interactions seem to induce immune responses to diseases, and in turn, the immune system affects the composition and diversity of the microbiome (Round and Mazmanian, 2009; Mangalmurti and Hunter, 2020; Attaway et al., 2021; Sun et al., 2022). COVID-19 has been reported to induce aberrant immune responses that not only elevate inflammatory markers including tumor necrosis factor-α, interleukin (IL)-10, and C-reactive protein, but also affect gut microbiome composition with a decreased population of beneficial bacteria especially bifidobacteria, *Eubacterium rectale*, and *Faecalibacterium prausnitzii* (Mangalmurti and Hunter, 2020; Attaway et al., 2021; Yeoh et al., 2021). Understanding microbiome changes across multiple microbiome compartments can shed light on the level and mechanisms of microbiome perturbation/dysbiosis associated with COVID-19, and may in turn aid the development of effective strategies for COVID-19 diagnosis, long-term management, and prevention.

All humans are susceptible to SARS-CoV-2 infection. Nevertheless, the human microbiome slightly differs across age, ethnicity, sex, race, and even geography (Human Microbiome Project Consortium, 2012; Brooks et al., 2018). Consequently, determining the effects of COVID-19 infections on the human microbiome requires assessing changes in the microbiomes of a wide range of patients across geographic regions. To this end, we performed a synthesis of human microbiome responses to COVID-19. We collected 16S rRNA gene amplicon sequence data from 11 studies sampling the oral and nasopharyngeal, or gut microbiome of COVID-19-infected and uninfected subjects. Our synthesis included 1,159 respiratory (oral and nasopharyngeal) microbiome samples and 267 gut microbiome samples and spanned four countries. We hypothesized that (1) because of stronger immune responses, infected patients would have a lower microbiome richness across microbiome compartments; (2) the microbiomes of infected patients would be more variable from host to host than that of healthy individuals, in line with the AKP; (3) as SARS-COV-2 is primarily a respiratory disease, the oral and nasopharyngeal microbiomes would be more strongly affected than the gut microbiomes; and (4) COVID-19 infection would result in consistent shifts in microbiome composition in both compartments.

## Materials and methods

### Literature search, selection criteria, and data extraction

**Figure 1:**
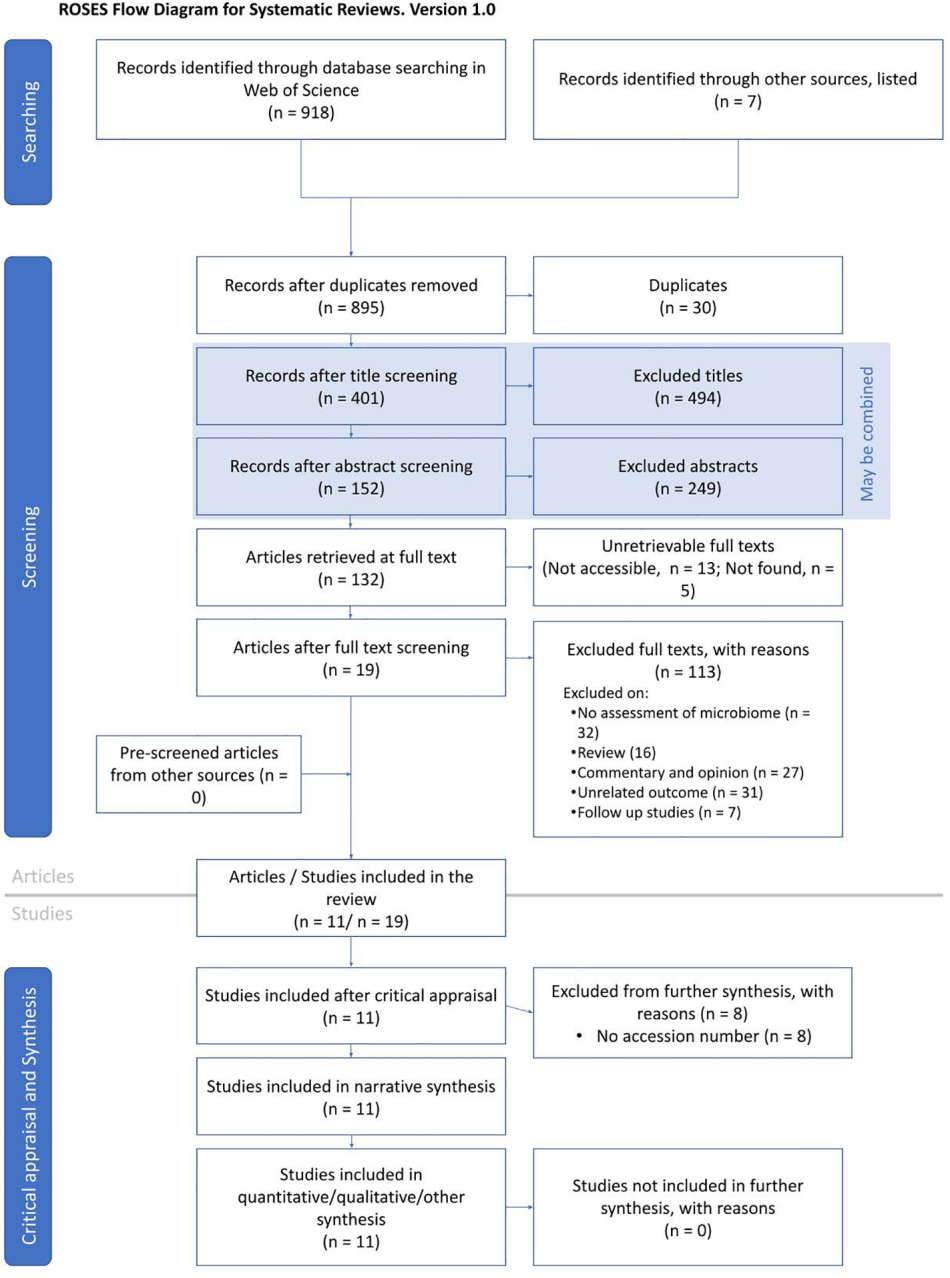
ROSES flowchart illustrating the systematic search, identification, screening, and final selection of articles.

This study was conducted through a robust literature search and selection using the RepOrting standards for Systematic Evidence Syntheses (ROSES) guidelines (Haddaway et al., 2018) (**Figure 1**). In January 2022, we performed a keyword search on the Web of Science database (www.webofscience.com) to identify and select relevant published articles. We used the terms “COVID-19” OR “SARS-CoV-2” OR “severe acute respiratory syndrome coronavirus 2” OR “coronavirus disease 2019” OR “nCoV” OR “novel coronavirus” AND “microbiome” OR “microbiota” OR “microflora” or “flora’’ OR “biome”. Furthermore, additional studies were included from other sources including PubMed and Google Scholar. We included published articles that performed 16S rRNA gene or transcript amplicon sequencing from gut/stool, nasopharyngeal, and oral samples collected from COVID-19+ individuals. We only included articles that were published in the English language. The authors independently reviewed the titles and abstracts of all the selected studies. Other COVID-19-related publications outside the scope of this study, as well as related commentaries, editorials, reviews, systematic reviews, and meta-analyses, were excluded.

For each selected study, we extracted information about the NCBI accession numbers to the 16S rRNA gene/transcript sequences, author names, patient characteristics, sample types, sample size, the 16S rRNA gene region sequenced, and the sequencing platform used. Studies were grouped based on the systems examined: gut microbiome, or oral, nasopharyngeal, and upper respiratory tract (URT) microbiomes (heretofore oral/URT).

### Bioinformatics and statistical analyses

All sequences were downloaded from NCBI as.fastq files and sequence data were processed using R (v 4.1) software (Bunn, 2008) and the *dada2* (Callahan et al., 2016) package. Following preliminary assessments of quality, we downloaded data for 1,588 samples from 11 studies. First, forward reads from each study were inspected to determine the optimal processing parameters using the *plotQualityProfile* function (detailed for each study in **Supplementary Table 1**), and trimmed to 100 base pairs with the *filterAndTrim* function, with *maxEE* = 2 and *truncQ* = 2. Reads were assigned a taxonomy using SILVA V.132 (Quast et al., 2013). The proportion of reads lost at each processing step for each study is shown in **Supplementary Figure S1**. According to available metadata, technical controls (e.g., blanks, mock communities) were removed prior to further processing.

Statistical analyses were performed with the *phyloseq* (McMurdie and Holmes, 2013), *vegan* (Oksanen et al., 2022), *Maaslin2* (Mallick et al., 2021), and *lmerTest* (Kuznetsova et al., 2017) packages. Prior to analyses, all samples were standardized to 2,000 reads per sample using the *rarefy_even_depth* function, which led to a loss of 63 samples. To assess the effects of COVID-19 infection on different compartments of microbial diversity (i.e., rare and dominant), we calculated Hill numbers (richness, effective Shannon diversity, and inverse Simpson diversity, or *q* = 0, *q* = 1, and *q* = 2, respectively; Chao et al., 2014). Richness is more heavily affected by the diversity of rare taxa, while inverse Simpson diversity is more affected by the diversity of dominant taxa. To determine the contribution of COVID-19 infection to oral/URT and gut microbiomes, we performed a distance-based variance partitioning analysis using the *varpart* function of *vegan*, with Bray–Curtis dissimilarities. The Bray–Curtis dissimilarities between samples taken from the same region among COVID-19-infected and uninfected patients were used to measure microbiome variance. Unless otherwise noted, diversity measures are presented as mean ± standard deviation.

To test the effect of COVID-19 infection on microbiome diversity (i.e., Hill numbers, H1) and variability (i.e., Bray–Curtis dissimilarities, H2), we used linear mixed effect models, with the study as a random effect, and COVID-19 infection as a fixed effect using the lmer function from the *lmerTest* package (Kuznetsova et al., 2017). To compare the effect of SARS-CoV-2 infection on gut and oral/URT microbiomes (H3), we used linear mixed effect models, with the study as a random effect, and the interaction between COVID-19 status and the sampled region (i.e., gut *vs.* oral/URT) as a fixed effect. In addition, a contrast analysis was performed using the emmeans function from the *emmeans* package (Lenth et al., 2023) to quantify the effect of COVID-19 infection within sampled regions. Model assumptions and performances were tested using the performance package (Lüdecke et al., 2021); all model outputs and performances are found in the **Supplementary Materials**.

To identify bacterial genera that were consistently under- or overrepresented in SARS-CoV-2-infected patients, we used microbiome-oriented linear models (*MaAsLin2* package; Mallick et al., 2021) for gut and oral/URT samples separately, with the study and sample type as random effects, SARS-CoV-2 infection as a fixed effect, and a prevalence threshold of 0.2.

## Results

In total, we collected 1,426 high-quality, processed samples from the USA (Chicago, Jackson, Nashville, New York City, Philadelphia, and San Diego), Spain (Alicante), France (Paris), and China (Guangdong, Shanghai, and Wuhan) (**Supplementary Figure S2**), represented by 29,491 amplicon sequence variants, or ASVs (**Supplementary Table 1**). Of the 11 studies surveyed, 8 included COVID-19-infected patients and controls (Engen et al., 2021; Merenstein et al., 2021; Minich et al., 2021; Newsome et al., 2021; Shilts et al., 2021; Smith et al., 2021; Ventero et al., 2021; Wu et al., 2021), and 2 of these (Newsome et al., 2021; Wu et al., 2021) also included samples of recovered patients. Three of the studies only sampled infected patients (Shilts et al., 2021; Xu et al., 2021; Ventero et al., 2022), and one sampled infected and recovered patients (Tian et al., 2021).

**Figure 2:**
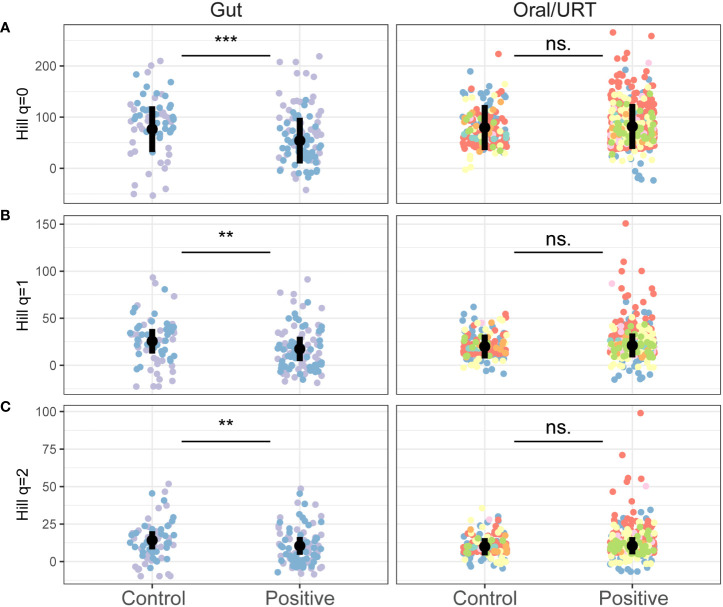
The effect of COVID-19 infection on the alpha diversity of the gut and oral/URT microbiomes. Hill richness (**A**: *q* = 0), effective Shannon (**B**: *q* = 1) and Inverse Simpson (**C**: *q* = 2) diversity indices were calculated to assess the impact of increasingly dominant portions of the community. Points are colored by studies, and the average across studies is shown with a black point. Significant differences between infected and non-infected patients across studies are indicated with asterisks where significant (***p < 0.001; ** *p* < 0.01) and with “n.s.” otherwise.

On average, gut microbiome samples were more diverse (136 ± 54 ASVs) than oral/URT samples (80 ± 79 ASVs). Within studies, COVID-19 infection caused minor, but consistent decreases in gut microbial richness (Hill *q* = 0, estimate ± SE = 22.46 ± 6.22, *p* < 0.001), but not in oral/URT richness (*p* = 0.55, **Figure 2**; **Supplementary Figure S3**). This decrease was also significant for *q* = 1 and *q* = 2 (*q* = 1: 8.20 ± 2.50, *p* = 0.001; *q* = 2: 3.71 ± 1.38, *p* = 0.008, **Figure 2**), highlighting richness losses in the dominant portion of the community.

**Figure 3:**
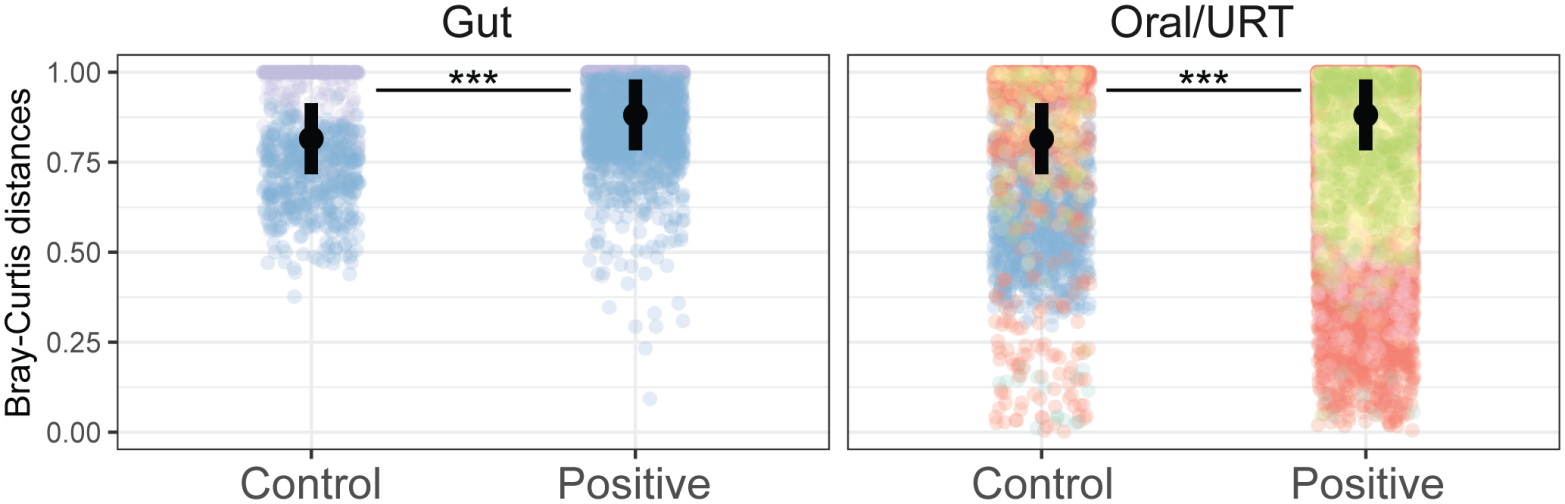
COVID-19 increases host-to-host variability across microbiomes. Community distances were measured as the Bray–Curtis distance between individuals in the same study, from the same body site, and with the same infection status. Points are colored by studies, and the average across studies is shown with a black point. Significant differences between infected and non-infected patients across studies are indicated with asterisks where significant (***p < 0.001).

SARS-CoV-2 infection led to changes in the microbiome composition, significantly explaining 2% of the variation in the microbial community, although these changes were study-dependent (*p* < 0.001, **Supplementary Figure S4**). Notably, of the 14.9% of the variance in community composition explained by each study, 7.3% could be ascribed to the participant’s country of origin (**Supplementary Figure S4**). In line with the AKP, patients infected with COVID-19 had a higher host-to-host variance in both the oral/URT and gut microbiome than uninfected patients (an increase of 0.06 ± 0.003 in Bray–Curtis dissimilarity relative to uninfected patients, *p* < 0.001, **Figure 3**).

**Figure 4:**
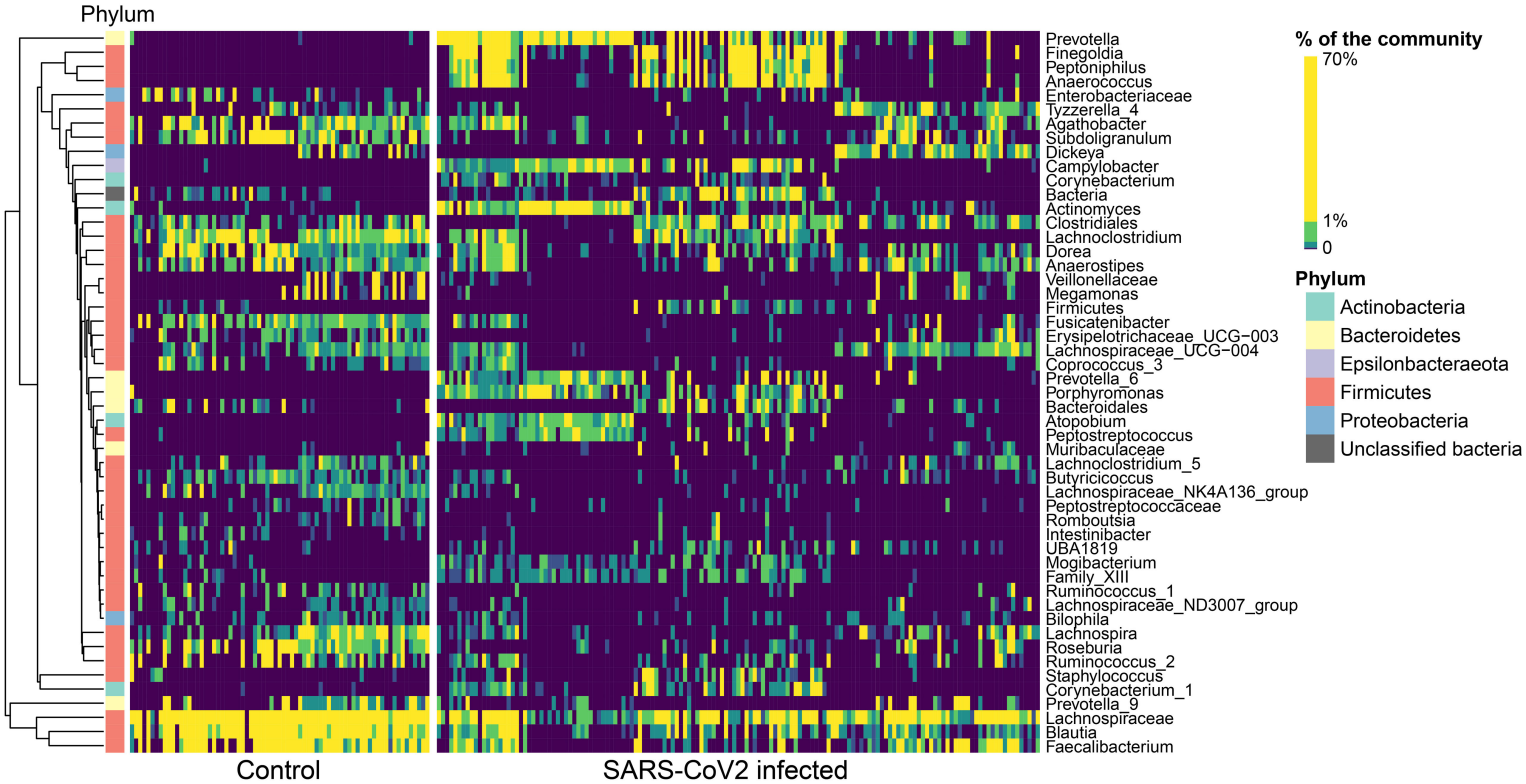
Altered relative abundances of bacterial genera in the gut microbiome of SARS-CoV-2-infected individuals. Bacterial genera that exhibit significantly different (*p* < 0.01) relative abundances between SARS-CoV- 2-infected and uninfected individuals in the gut microbiome were selected using the MaAsLin2 approach, which included random effects for sample type and study. Only significantly different genera are displayed, relative abundances are colored by quantiles, and genera are grouped according to Ward’s clustering method. Phylum membership is displayed on the left bar. These 51 genera make up 38.3% ± 22.2% of the community, on average across all samples.

**Figure 5:**
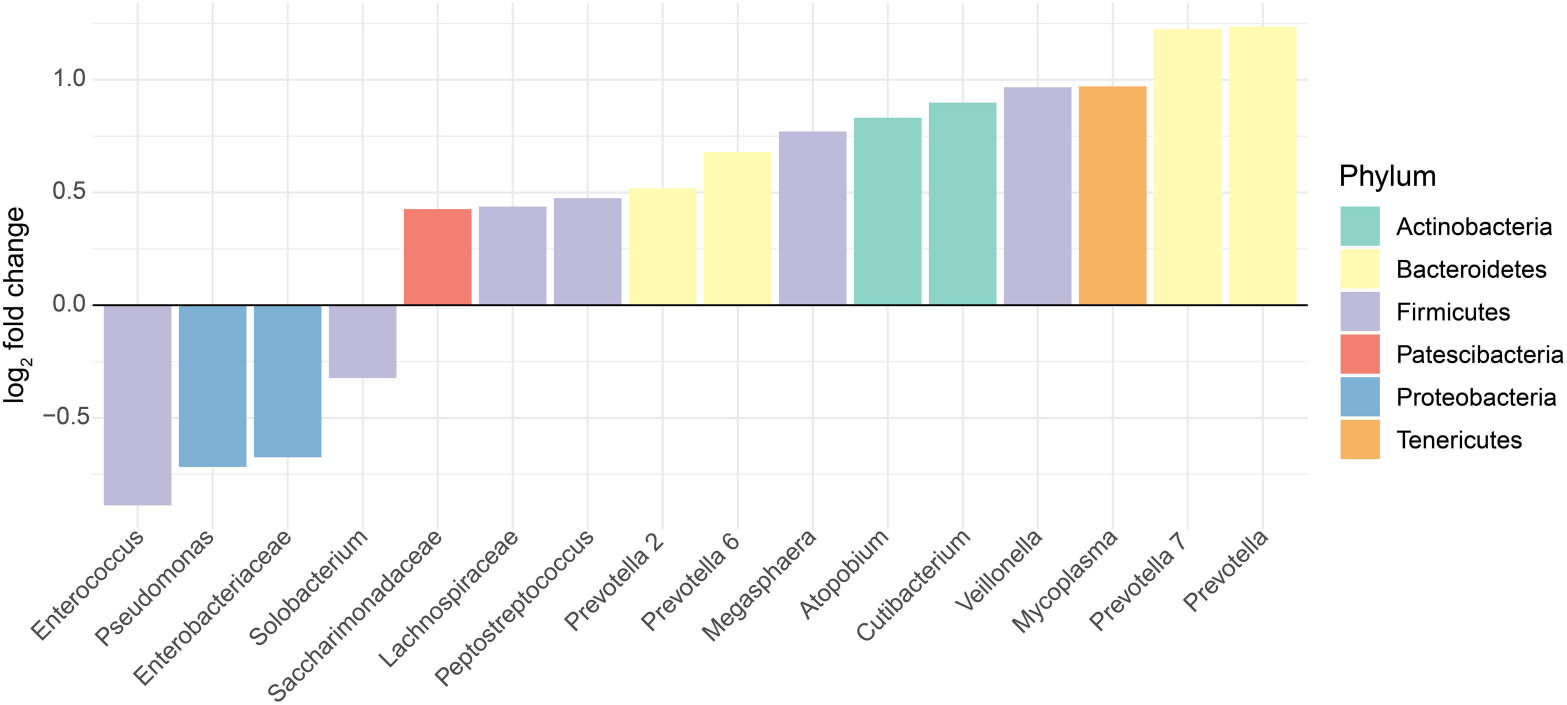
Altered relative abundances of bacterial genera in the oral and URT microbiome of SARS-CoV-2-infected individuals. Bacterial genera that exhibit significantly different (*p* < 0.01) relative abundances between SARS-CoV-2-infected and uninfected individuals in the oral or URT microbiome were selected using the MaAsLin2 approach, which included random effects for sample type and study. The log_2_ fold changes in taxon abundances for infected patients relative to non-infected patients are displayed. Only genera with significantly different (*p* < 0.01) abundances between these two groups are displayed, and in total, they represent 23.8% ± 25.9% of the whole community, on average across all samples.

In general, the gut microbiomes of infected and non-infected patients had more consistent shifts across studies for the gut than for the oral/URT microbiomes. We identified 51 dominant genera (38.3% ± 22.2% of the community) in the gut microbiome, whose relative abundances consistently and significantly differed between infected and non-infected patients across studies (**Figure 4**). These taxa belonged predominantly to Firmicutes, with the most consistent decreases in *Fusicatenibacter, Lachnospiraceae NK4A316 group, Lachnoclostridum, Blautia*, and *Roseburia* and the most consistent increases in *Finegoldia, Porphyromonas, Anaerococcus*, and *Peptoniphilus* for COVID-19-infected patients, relative to uninfected patients. In contrast, we only found 16 genera (23.8% ± 25.9% of the community on average) that consistently differed between the oral/URT microbiome of COVID-19-infected and uninfected patients (**Figure 5**). Of these, *Enterococcus, Pseudomonas, unclassified Enterobacteriaceae*, and *Solobacterium* were lower in uninfected patients, whereas *Prevotella, Mycoplasma, Veillonella, Cutibacterium, Atopobium*, and *Megasphaera* were consistently and significantly more abundant in COVID-19-infected patients.

## Discussion

SARS-CoV-2 invades the human body mainly through the angiotensin-converting enzyme 2 (ACE2) and cofactor transmembrane serine protease 2 (TMPRSS2) receptors in the epithelial cells of the nasopharyngeal tract, and then gradually moves to initiate infection in the lungs, which gradually results in gastrointestinal involvement as well as affects other organs including the heart, kidneys, pancreas, eyes, and skin (Gavriatopoulou et al., 2020; Hoque et al., 2021a; Rahman et al., 2021; Rahman et al., 2021). Interestingly, high levels of both ACE2 and TMPRSS2 receptors are naturally expressed in multiple organs of the human respiratory and gastrointestinal tracts (Perlot & Penninger, 2013; Roncon et al., 2020; Xiao et al., 2020), thus enabling SARS-CoV-2 to circulate and induce severe inflammation, immune imbalance, and microbiome dysbiosis within these systems (Hoffmann et al., 2020; Villapol, 2020). Moreover, recent insights into coronavirus biology and SARS-CoV-2-human interactions attribute COVID-19 pathophysiology to aberrant and aggressive immune responses in SARS-CoV-2 clearance (Hoque et al., 2021b; Yeoh et al., 2021). COVID-19 infection can therefore result in a wide variety of responses in the human-associated microbiome, which may be further modulated by the host’s environment, such as diet and exposure to pollutants, which is population-specific. By simultaneously reanalyzing microbiome data from various microbiome compartments in infected and uninfected patients across the world, we sought to identify consistent, COVID-19 infection-specific changes in the human microbiome.

Consistent with our hypotheses, we found that SARS-CoV-2-infected individuals had a lower microbial diversity in the gut, but not in the upper respiratory tract. The reduction of gut microbial diversity in COVID-19 has been similarly reported (Zuo et al., 2020; Mazzarelli et al., 2021; Tian et al., 2021), regardless of antibiotic use (Zuo et al., 2020; Mazzarelli et al., 2021) and even several weeks after viral clearance (Zuo et al., 2020; Tian et al., 2021; Xu et al., 2021). The gut microbiome is relatively stable, and a major predictor of normal gut functioning, immunomodulation, and overall host health (Rooks and Garrett, 2016; Tian et al., 2021; Xu et al., 2021). Our study highlights SARS-CoV-2’s ability to disrupt human gut microbiome eubiosis through the depletion of gut microbial diversity, which may contribute to disease severity and opportunistic infections. The consistent decrease in diversity found across Hill numbers for the gut microbiome richness highlights that richness loss occurs in dominant taxa. This may have major implications on the composition and diversity of the gut microbiome in COVID-19 infection resulting in dysbiosis, impaired immune functioning, pro-inflammatory conditions, etc.

Our study did not control for COVID-19 patients’ medication use (e.g., antibiotics and antivirals), age, genetic background, sex, or diet, which may also affect the gut microbial diversity and further confound COVID-19-associated gut microbial signatures. However, the consistent results we recorded across studies after controlling for study–study particularities suggest that the decrease in gut microbial diversity is indeed due to SARS-CoV-2 infection. Intriguingly, the intestinal ACE2, which is the receptor of SARS-CoV-2, plays a vital role in maintaining the gut microbiome eubiosis (Hamming et al., 2004; Hashimoto et al., 2012; Tian et al., 2021), and the SARS-CoV-2 infection may downregulate the expression and availability of ACE2, which could disrupt gut homeostasis, adversely impacting microbial diversity. Our inability to detect a significant effect on the microbial richness of the oral/URT microbiome may be attributed to the oral/URT microbiome being more dynamic, resilient, and transient than the gut microbiome due to frequent bidirectional air and mucus movement as well as its regular exposure to the environment (Huffnagle et al., 2017). Recent studies have found inconsistent effects of COVID-19 on the microbial diversity of the URT (De Maio et al., 2020; Mostafa et al., 2020; Braun et al., 2021; Miao et al., 2021; Yamamoto et al., 2021; Zhang et al., 2021; Rafiqul Islam et al., 2022; Uehara et al., 2022), and others have proposed that SARS-CoV-2 has weak effects on the URT microbiome (De Maio et al., 2020; Braun et al., 2021; Yamamoto et al., 2021) akin to acute respiratory virus infections in humans (Ramos-Sevillano et al., 2019; De Maio et al., 2020; Yamamoto et al., 2021).

Higher variability is generally associated with lower stability and predictability. In accordance with previous studies (Ma, 2020; Altabtbaei et al., 2021), we also found that host-to-host gut and URT microbiome variability was greater in SARS-CoV-2-infected patients than in non-infected controls, in line with the AKP (Zaneveld et al., 2017) and with a previous synthesis, which found that most human-associated diseases result in a higher microbiome variability across patients (Ma, 2020).

Notably, our study shows that COVID-19 infection resulted in an overall loss of beneficial bacteria, and worryingly, a consistent increase in pathogenic bacteria, particularly in the oral/URT microbiome. The increased relative abundances and colonization of opportunistic pathogens including *Mycoplasma*, *Prevotella*, *Peptostreptococcus*, *Veillonella*, *Cutibacterium*, and Saccharibacteria in the oral/URT recorded in our study may be associated with the early-stage SARS-CoV-2-induced inflammation, the loss of beneficial bacteria, and the increased exposure and receptiveness to allochthonous and indigenous microorganisms (Man et al., 2017; Dubourg et al., 2019).

In the gut microbiome, increased relative abundances of pathogenic bacteria including *Campylobacter*, *Corynebacterium*, *Staphylococcus*, *Clostridium*, *Peptostreptococcus*, *Prevotella*, *Anaerococcus*, *Actinomyces*, *Porphyromonas*, and *Bacteroides* were recorded in SARS-CoV-2 infection. Increasingly, emerging reports posit that alterations in the gut microbiome may facilitate blooms of both pathogenic and previously rare bacteria, which can further aggravate overall gut inflammation (Mazzarelli et al., 2021; Tian et al., 2021; Xu et al., 2021). The presence and increased abundance of common oral/URT commensals and pathogens in the gut (e.g., *Corynebacterium*, *Peptostreptococcus*, *Porphyromonas*, *Prevotella*, and *Staphylococcus*) may suggest a possible translocation of these organisms from the oral/URT to the gut. Previously, inflammation, disruption, and increased permeability of membrane mucosa were associated with COVID-19 (Cao and Li, 2020). Increased permeability of membrane mucosa facilitates the translocation of some oral/URT microbes as well as enriched opportunistic pathogens to the gut (Man et al., 2017; Cao and Li, 2020; Giron et al., 2021).

We also detected the loss of beneficial microbes *Fusicatenibacter, Lachnospiraceae NK4A316 group, Lachnoclostridium, Blautia*, and *Roseburia* in the gut. These beneficial bacteria often enhance and maintain the integrity and function of mucosal barriers, metabolism, and immunomodulation, and protect against pathogen invasion through several mechanisms including the secretion of short-chain fatty acids (SCFA) and antimicrobial peptides (Gallo and Hooper, 2012; Abt & Pamer, 2014; Zhang et al., 2015). In line with our findings, SARS-CoV-2-associated microbiome perturbations were previously associated with a decline in SCFA (Lv et al., 2021; Sokol et al., 2021), thus promoting a systemic pro-inflammatory condition (Qin et al., 2015; Esquivel-Elizondo et al., 2017) and the severity of pulmonary viral infections such as COVID-19 (Chemudupati et al., 2020; Friedland and Haribabu, 2020; Tang et al., 2020). In the same vein, Lv and colleagues reported a pathogen-regulated feedback loop between the decline in SCFA production and SARS-CoV-2 infection (Lv et al., 2021).

Whether increased abundances of gut and oral/URT pathogenic and pro-inflammatory bacteria in SARS-CoV-2 infection actually play an active part in COVID-19 or mainly thrive opportunistically, exploiting the depletion of commensal bacteria, remains unknown. Nevertheless, our findings demonstrate that oral/URT and gut microbiomes are systematically perturbed by COVID-19, resulting in a lower microbial diversity, loss of beneficial microbes, and increased presence of pathogenic bacteria, which could trigger prolonged pro-inflammatory reactions, immunological changes, and secondary bacterial infections that could account for chronic COVID-19-associated symptoms, as well as prolonged sequelae. Understanding the dynamics of COVID-19-associated microbiome alterations may help identify microbiome-based strategies with potential applications in COVID-19 management and treatment. Furthermore, our findings highlight that non-invasive organ and/or system-based microbiome profiling may serve not only for COVID-19 diagnosis and prognosis but also, for the identification of individuals at risk of secondary infections, chronic disease, and/or degenerative inflammatory symptoms, including Kawasaki-like disease (KLD) and multisystem inflammation, as is the case with children and young adults (Akca et al., 2020; Cheung et al., 2020; Sokolovsky et al., 2021; Elouardi et al., 2022).

Finally, human microbiome composition and diversity are highly heterogeneous and largely driven by biogeographies, environments, ethnicity, and socioeconomic status (Amato et al., 2021; Yamamoto et al., 2021; Yeoh et al., 2021). While our study design highlights the importance of sampling across human populations to understand a disease, our study lacks samples from the Southern Hemisphere, in line with recent reports indicating very limited public human microbiome data from the Global South (Abdill et al., 2022). A global representation of data in human microbiome studies is critical to understanding global drivers and patterns of disease (in this case, COVID-19) to provide sustainable interventions to all populations without bias.

## Data availability statement

INSDC accession numbers for each dataset reused in this study are included in **Supplementary Table 1**. Code used for data analysis is available in https://github.com/drcarrot/COVID, and detailed model descriptions are included in the **Supplementary Material**.

## Author contributions

RR conceptualized the study, carried out the literature search, reviewed and selected relevant literature, and wrote the article. RB performed the statistical analyses. SJ conceptualized the study, extracted and assembled the 16S rRNA data and metadata, organized the presentation of the results, and created all figures. All authors contributed substantially to subsequent revisions. All authors approved the submitted version.
