# Supplementary material for: COVID-19 alters human microbiomes: a meta-analysis
Source: Front Cell Infect Microbiol. 2023 Aug 2;13:1211348. doi: 10.3389/fcimb.2023.1211348 (PMC10433767; doi:10.3389/fcimb.2023.1211348)
Supplement: Supplementary file 1 [file DataSheet_1.pdf]

Suppl. model fits

Rine Christopher Reuben, Rémy Beugnon and Stephanie Jurburg

2023-01-23

Contents

1 Hypothesis I **3**

1.1 Effect of COVID-19 on microbiomes 3

1.1.1 Richness 3

1.1.1.1 Model fit quality 3

1.1.1.2 Model fit summary 3

1.1.2 Shannon 4

1.1.2.1 Model fit quality 4

1.1.2.2 Model fit summary 5

1.1.3 Simpson 6

1.1.3.1 Model fit quality 6

1.1.3.2 Model fit summary 6

2 Hypothesis II **8**

2.1 Effect of COVID-19 on microbiomes distances 8

2.1.0.1 Model fit quality 8

2.1.0.2 Model fit summary 8

3 Hypothesis III **9**

3.1 Effect of COVID-19 per microbiome region (gut vs. oral/URT) 9

3.1.1 Hill q=0 9

3.1.1.1 Model fit quality 9

3.1.1.2 Model fit summary 9

3.1.2 Hill q=1 11

3.1.2.1 Model fit quality 11

3.1.2.2 Model fit summary 11

3.1.3 Hill q=2 12

3.1.3.1 Model fit quality 12

3.1.3.2 Model fit summary . . . 13
3.1.4 Community distances . . . 14
3.1.4.1 Model fit quality . . . 14
3.1.4.2 Model fit summary . . . 14
3.2 Effect of COVID per sampling method . . . 16
3.2.1 Hill q=0 . . . 16
3.2.1.1 Model fit quality . . . 17
3.2.1.2 Model fit summary . . . 17
3.2.2 Hill q=1 . . . 21
3.2.2.1 Model fit quality . . . 21
3.2.2.2 Model fit summary . . . 21
3.2.3 Hill q=2 . . . 25
3.2.3.1 Model fit quality . . . 25
3.2.3.2 Model fit summary . . . 25
3.2.4 Community distances . . . 29
3.2.4.1 Model fit quality . . . 29
3.2.4.2 Model fit summary . . . 29
3.2.4.3 Hypothesis IV . . . 30

1 Hypothesis I

.

1.1 Effect of COVID-19 on microbiomes

.

1.1.1 Richness

.

1.1.1.1 Model fit quality .

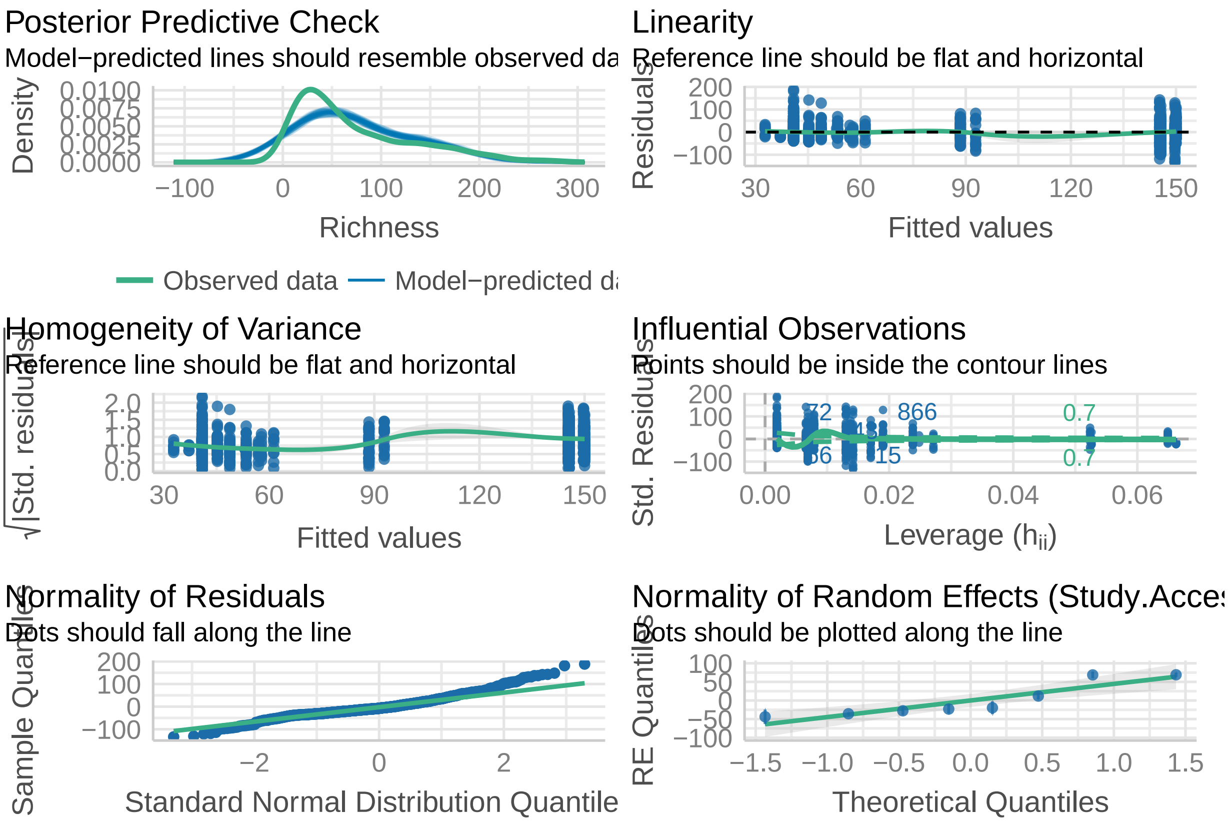

.

1.1.1.2 Model fit summary .

```
Linear mixed model fit by REML. t-tests use Satterthwaite's method [
lmerModLmerTest]
Formula: "Richness ~ COVID + (1|Study.Accession)"
   Data: df.1
```

```
REML criterion at convergence: 10377.5

Scaled residuals:
    Min      1Q  Median      3Q     Max
-3.3820 -0.6039 -0.1756  0.4917  4.7593

Random effects:
 Groups          Name        Variance Std.Dev.
 Study.Accession (Intercept) 2113     45.96
 Residual                    1563     39.53
Number of obs: 1016, groups:  Study.Accession, 8

Fixed effects:
            Estimate Std. Error       df t value Pr(>|t|)
(Intercept)   80.888     16.543    7.314   4.890  0.00157 **
COVIDYes      -4.363      3.151 1008.717  -1.385  0.16644
---
Signif. codes:  0 '***' 0.001 '**' 0.01 '*' 0.05 '.' 0.1 ' ' 1

Correlation of Fixed Effects:
         (Intr)
COVIDYes -0.133

Type III Analysis of Variance Table with Satterthwaite's method
      Sum Sq Mean Sq NumDF  DenDF F value Pr(>F)
COVID 2996.1  2996.1     1 1008.7  1.9175 0.1664
```

1.1.2 Shannon

.

.

1.1.2.1 Model fit quality .

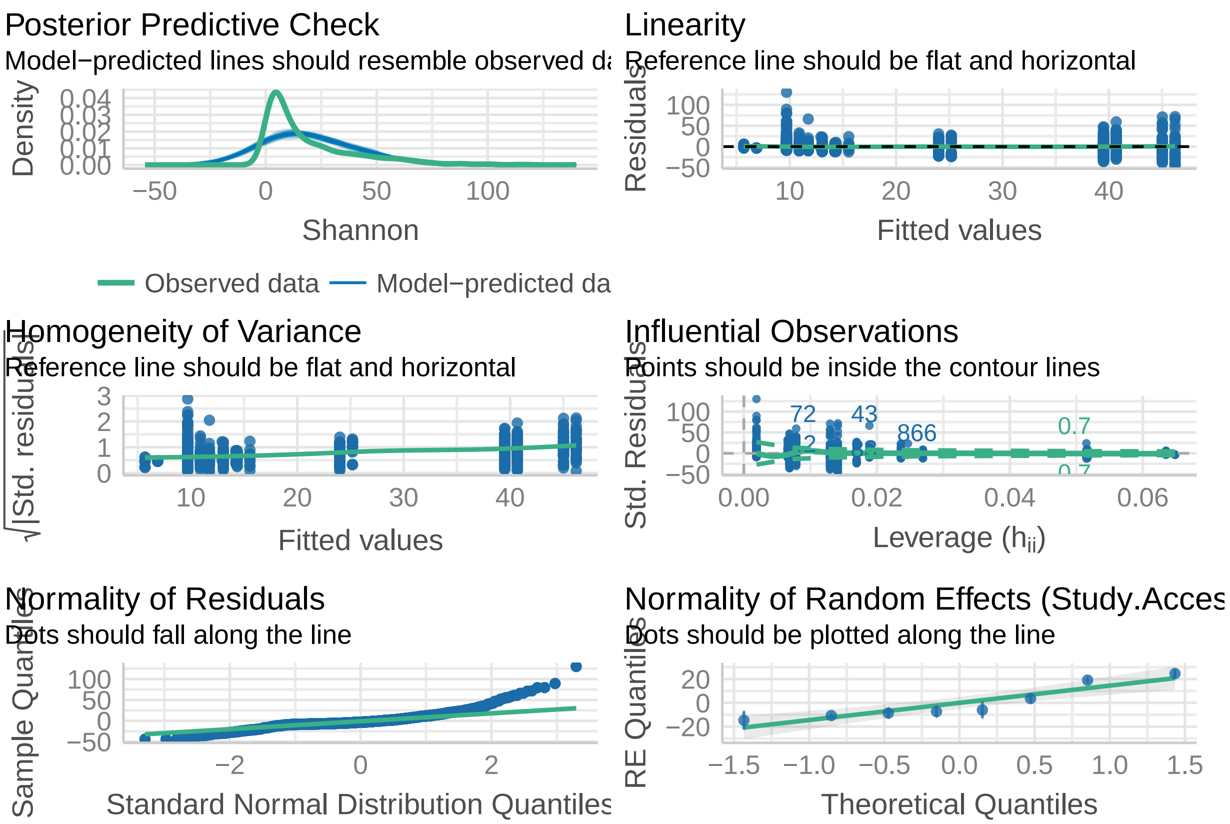

1.1.2.2 Model fit summary .

```
Linear mixed model fit by REML. t-tests use Satterthwaite's method [
lmerModLmerTest]
Formula: "Shannon ~ COVID + (1|Study.Accession)"
   Data: df.1

REML criterion at convergence: 8513.3

Scaled residuals:
    Min      1Q  Median      3Q     Max
-2.7912 -0.4712 -0.2165  0.3366  8.2426

Random effects:
 Groups          Name        Variance Std.Dev.
 Study.Accession (Intercept) 217.3    14.74
 Residual                    249.3    15.79
Number of obs: 1016, groups:  Study.Accession, 8

Fixed effects:
            Estimate Std. Error       df t value Pr(>|t|)
(Intercept)   21.572      5.356    7.461   4.028  0.00439 **
COVIDYes      -1.191      1.258 1009.518  -0.947  0.34405
---
Signif. codes:  0 '***' 0.001 '**' 0.01 '*' 0.05 '.' 0.1 ' ' 1
```

```
Correlation of Fixed Effects:
         (Intr)
COVIDYes -0.165
```

```
Type III Analysis of Variance Table with Satterthwaite's method
      Sum Sq Mean Sq NumDF  DenDF F value Pr(>F)
COVID 223.36  223.36     1 1009.5  0.8961  0.344
```

.

1.1.3 Simpson

.

1.1.3.1 Model fit quality .

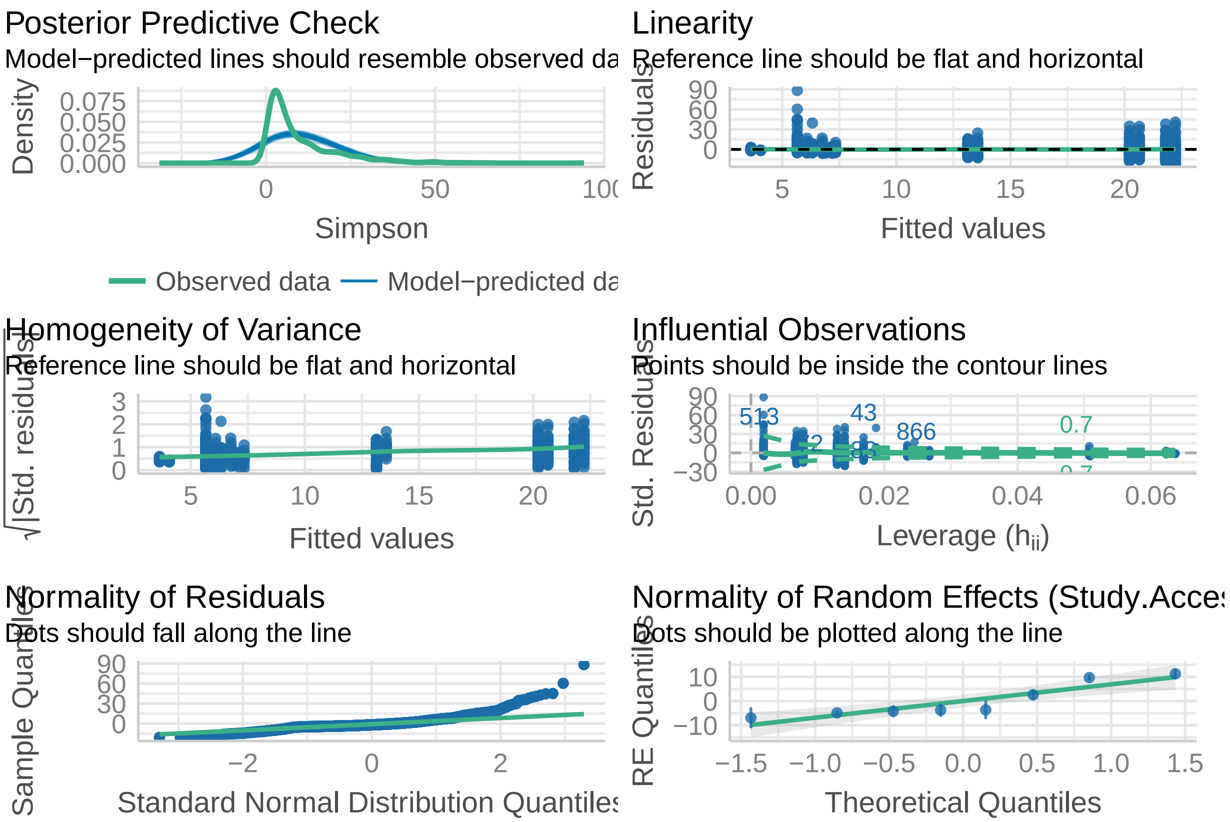

.

1.1.3.2 Model fit summary .

```
Linear mixed model fit by REML. t-tests use Satterthwaite's method [
lmerModLmerTest]
Formula: "Simpson ~ COVID + (1|Study.Accession)"
```

```
   Data: df.1

REML criterion at convergence: 7310.1

Scaled residuals:
    Min      1Q  Median      3Q     Max
-2.3701 -0.4614 -0.2124  0.2518 10.1189

Random effects:
 Groups          Name        Variance Std.Dev.
 Study.Accession (Intercept) 50.90    7.134
 Residual                    76.23    8.731
Number of obs: 1016, groups:  Study.Accession, 8

Fixed effects:
             Estimate Std. Error        df t value Pr(>|t|)
(Intercept)   10.9824     2.6128    7.5918   4.203  0.00335 **
COVIDYes      -0.4271     0.6957 1010.1444  -0.614  0.53942
---
Signif. codes:  0 '***' 0.001 '**' 0.01 '*' 0.05 '.' 0.1 ' ' 1

Correlation of Fixed Effects:
         (Intr)
COVIDYes -0.187

Type III Analysis of Variance Table with Satterthwaite's method
      Sum Sq Mean Sq NumDF  DenDF F value Pr(>F)
COVID 28.728  28.728     1 1010.1  0.3769 0.5394
```

2 Hypothesis II

.

2.1 Effect of COVID-19 on microbiomes distances

.

2.1.0.1 Model fit quality .

2.1.0.2 Model fit summary .

```
Linear mixed model fit by REML. t-tests use Satterthwaite's method [
lmerModLmerTest]
Formula: "Dist ~ COVID + (1|Study.Accession)"
   Data: df.dist

REML criterion at convergence: -59590.8

Scaled residuals:
    Min      1Q  Median      3Q     Max
-5.9781 -0.2420  0.3939  0.6103  2.9376

Random effects:
 Groups          Name        Variance Std.Dev.
 Study.Accession (Intercept) 0.02559  0.1600
 Residual                    0.02256  0.1502
Number of obs: 62546, groups:  Study.Accession, 8

Fixed effects:
             Estimate Std. Error        df t value Pr(>|t|)
(Intercept) 7.122e-01  5.672e-02 6.931e+00   12.56 5.08e-06 ***
COVIDYes    5.598e-02  2.962e-03 6.254e+04   18.90  < 2e-16 ***
---
Signif. codes:  0 '***' 0.001 '**' 0.01 '*' 0.05 '.' 0.1 ' ' 1

Correlation of Fixed Effects:
         (Intr)
COVIDYes -0.041


Type III Analysis of Variance Table with Satterthwaite's method
      Sum Sq Mean Sq NumDF DenDF F value    Pr(>F)
COVID 8.0543  8.0543     1 62544  357.07 < 2.2e-16 ***
---
Signif. codes:  0 '***' 0.001 '**' 0.01 '*' 0.05 '.' 0.1 ' ' 1
```

3 Hypothesis III

.

3.1 Effect of COVID-19 per microbiome region (gut vs. oral/URT)

.

3.1.1 Hill q=0

.

3.1.1.1 Model fit quality .

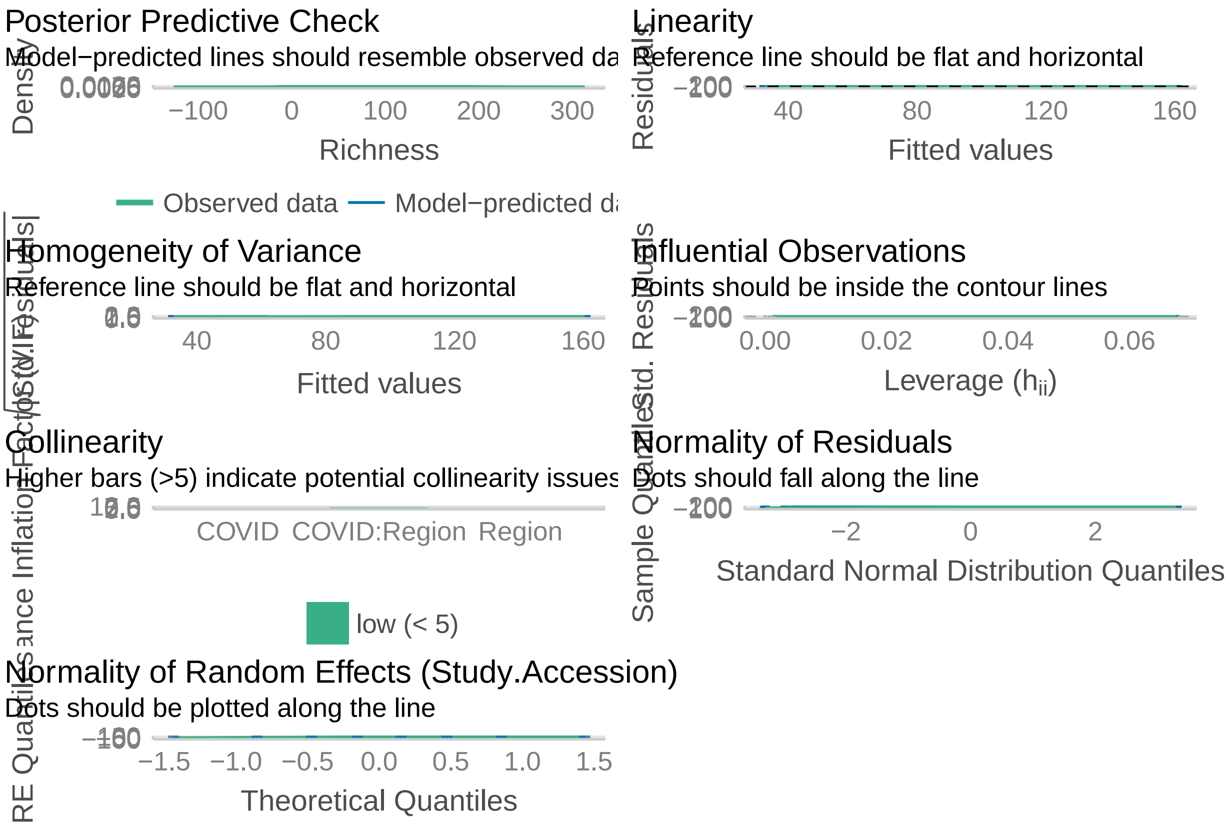

3.1.1.2 Model fit summary .

```
Linear mixed model fit by REML. t-tests use Satterthwaite's method [
lmerModLmerTest]
Formula: "Richness ~ COVID * Region + (1|Study.Accession)"
   Data: df.1

REML criterion at convergence: 10346.1
```

```
Scaled residuals:
    Min      1Q  Median      3Q     Max
-3.6899 -0.6131 -0.1716  0.4875  4.7853

Random effects:
 Groups          Name        Variance Std.Dev.
 Study.Accession (Intercept) 2731     52.26
 Residual                    1532     39.14
Number of obs: 1016, groups:  Study.Accession, 8

Fixed effects:
                        Estimate Std. Error       df t value Pr(>|t|)
(Intercept)               76.461     19.590    8.484   3.903 0.004037 **
COVIDYes                 -22.457      6.221 1004.948  -3.610 0.000321 ***
RegionOral/URT             3.097      7.310 1011.940   0.424 0.671877
COVIDYes:RegionOral/URT   24.594      7.192 1005.402   3.420 0.000652 ***
---
Signif. codes:  0 '***' 0.001 '**' 0.01 '*' 0.05 '.' 0.1 ' ' 1

Correlation of Fixed Effects:
            (Intr) COVIDYs RO/URT
COVIDYes    -0.193
RegnOrl/URT -0.293  0.519
COVIDY:RO/U  0.154 -0.865  -0.586

Type III Analysis of Variance Table with Satterthwaite's method
             Sum Sq Mean Sq NumDF  DenDF F value    Pr(>F)
COVID         12228   12228     1 1005.5  7.9828 0.0048158 **
Region        10202   10202     1 1008.2  6.6603 0.0099986 **
COVID:Region  17914   17914     1 1005.4 11.6948 0.0006519 ***
---
Signif. codes:  0 '***' 0.001 '**' 0.01 '*' 0.05 '.' 0.1 ' ' 1

$`simple contrasts for COVID`
Region = Gut:
 contrast estimate   SE   df t.ratio p.value
 No - Yes    22.46 6.22 1005   3.610  0.0003

Region = Oral/URT:
 contrast estimate   SE   df t.ratio p.value
 No - Yes    -2.14 3.61 1007  -0.592  0.5539

Degrees-of-freedom method: kenward-roger

$`simple contrasts for Region`
COVID = No:
 contrast         estimate   SE   df t.ratio p.value
 Gut - (Oral/URT)     -3.1 7.33 1012  -0.423  0.6725

COVID = Yes:
 contrast         estimate   SE   df t.ratio p.value
 Gut - (Oral/URT)    -27.7 6.62 1010  -4.182  <.0001

Degrees-of-freedom method: kenward-roger
```

3.1.2 Hill q=1

.

3.1.2.1 Model fit quality .

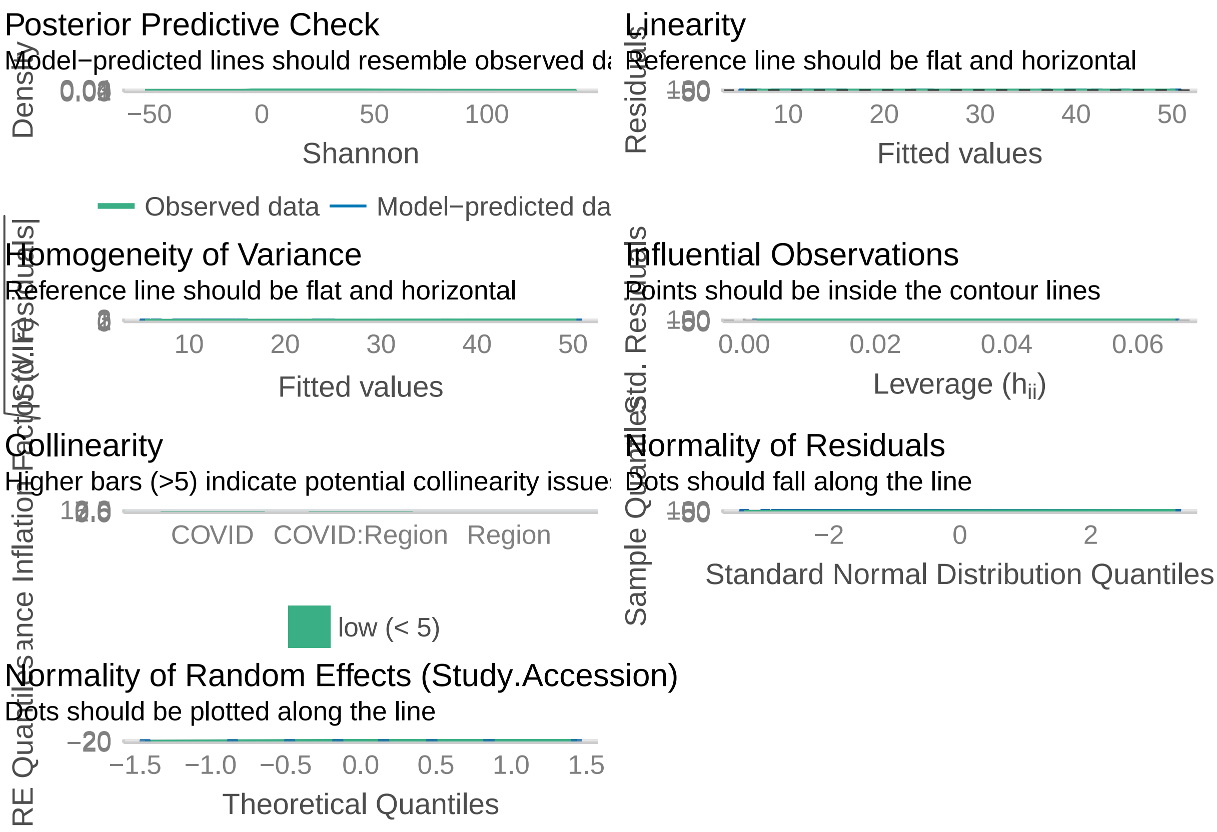

3.1.2.2 Model fit summary .

```
Linear mixed model fit by REML. t-tests use Satterthwaite's method [
lmerModLmerTest]
Formula: "Shannon ~ COVID * Region + (1|Study.Accession)"
   Data: df.1

REML criterion at convergence: 8495.2

Scaled residuals:
    Min      1Q  Median      3Q     Max
-3.0671 -0.4684 -0.2163  0.3260  8.2584

Random effects:
 Groups          Name        Variance Std.Dev.
 Study.Accession (Intercept) 222.2    14.91
 Residual                    247.1    15.72
Number of obs: 1016, groups:  Study.Accession, 8
```

```
Fixed effects:
                        Estimate Std. Error       df t value Pr(>|t|)   
(Intercept)               25.550      5.878    9.897   4.346  0.00149 **
COVIDYes                  -8.202      2.499 1004.872  -3.283  0.00106 **
RegionOral/URT            -5.645      2.925 1003.013  -1.930  0.05392 . 
COVIDYes:RegionOral/URT    9.370      2.888 1005.753   3.244  0.00122 **
---
Signif. codes:  0 '***' 0.001 '**' 0.01 '*' 0.05 '.' 0.1 ' ' 1

Correlation of Fixed Effects:
            (Intr) COVIDYs RO/URT
COVIDYes    -0.258               
RegnOrl/URT -0.389  0.520        
COVIDY:RO/U  0.206 -0.865  -0.588
```

```
Type III Analysis of Variance Table with Satterthwaite's method
              Sum Sq Mean Sq NumDF   DenDF F value   Pr(>F)   
COVID        1466.06 1466.06     1 1005.89  5.9324 0.015037 * 
Region         40.16   40.16     1  972.46  0.1625 0.686932   
COVID:Region 2600.71 2600.71     1 1005.75 10.5239 0.001217 **
---
Signif. codes:  0 '***' 0.001 '**' 0.01 '*' 0.05 '.' 0.1 ' ' 1
```

```
$`simple contrasts for COVID`
Region = Gut:
 contrast estimate   SE   df t.ratio p.value
 No - Yes     8.20 2.50 1005   3.283  0.0011

Region = Oral/URT:
 contrast estimate   SE   df t.ratio p.value
 No - Yes    -1.17 1.45 1008  -0.805  0.4208

Degrees-of-freedom method: kenward-roger 

$`simple contrasts for Region`
COVID = No:
 contrast         estimate   SE   df t.ratio p.value
 Gut - (Oral/URT)     5.65 2.94 1003   1.922  0.0549

COVID = Yes:
 contrast         estimate   SE   df t.ratio p.value
 Gut - (Oral/URT)    -3.72 2.65  986  -1.403  0.1608

Degrees-of-freedom method: kenward-roger 
```

3.1.3 Hill q=2

.

3.1.3.1 Model fit quality .

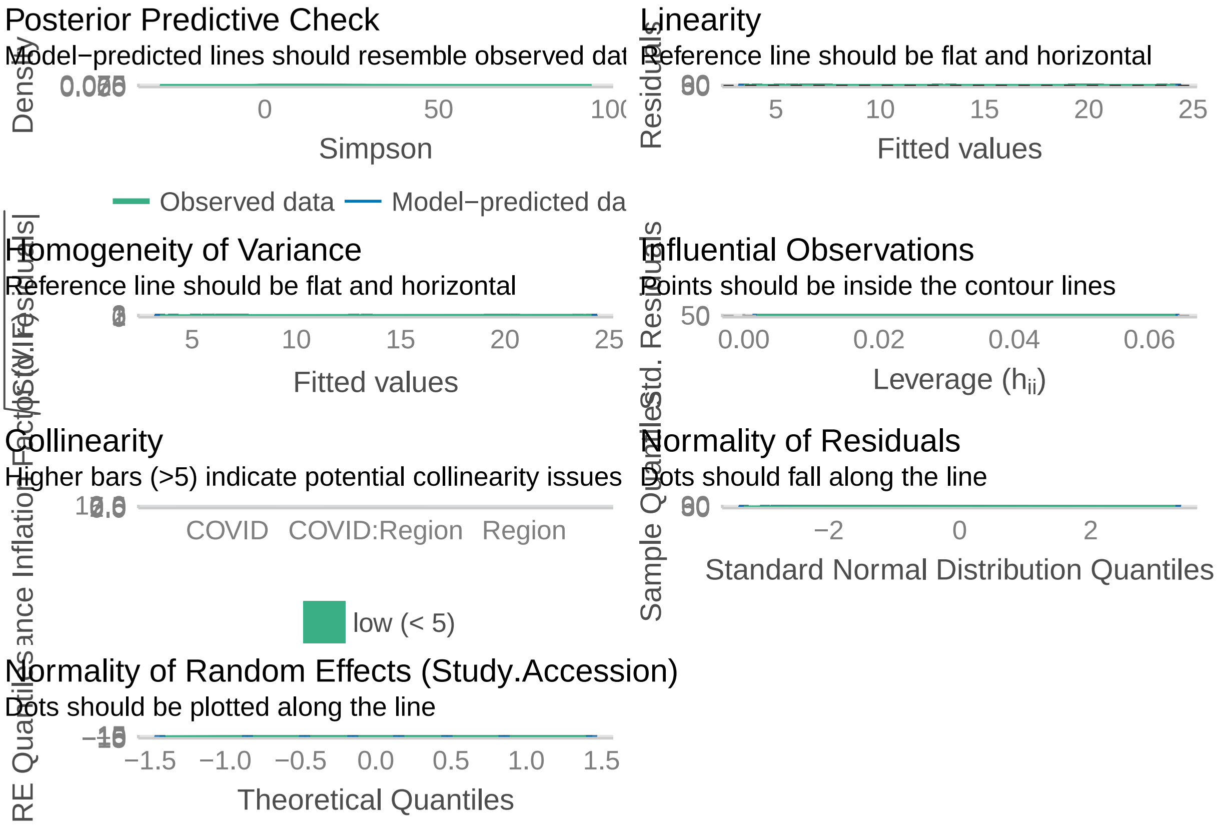

3.1.3.2 Model fit summary .

```
Linear mixed model fit by REML. t-tests use Satterthwaite's method [
lmerModLmerTest]
Formula: "Simpson ~ COVID * Region + (1|Study.Accession)"
   Data: df.1

REML criterion at convergence: 7296

Scaled residuals:
    Min      1Q  Median      3Q     Max
-2.5996 -0.4549 -0.2091  0.2834 10.1331

Random effects:
 Groups          Name        Variance Std.Dev.
 Study.Accession (Intercept) 44.69    6.685
 Residual                    75.77    8.705
Number of obs: 1016, groups:  Study.Accession, 8

Fixed effects:
                        Estimate Std. Error       df t value Pr(>|t|)
(Intercept)               14.186      2.765   11.412   5.131 0.000292 ***
COVIDYes                  -3.707      1.384 1004.834  -2.680 0.007491 **
RegionOral/URT            -4.248      1.614  978.965  -2.632 0.008622 **
COVIDYes:RegionOral/URT    4.345      1.599 1006.140   2.717 0.006702 **
```

```
---
Signif. codes:  0 '***' 0.001 '**' 0.01 '*' 0.05 '.' 0.1 ' ' 1

Correlation of Fixed Effects:
            (Intr) COVIDYs RO/URT
COVIDYes    -0.304
RegnOrl/URT -0.456  0.522
COVIDY:RO/U  0.243 -0.865  -0.591


Type III Analysis of Variance Table with Satterthwaite's method
             Sum Sq Mean Sq NumDF   DenDF F value   Pr(>F)
COVID        279.24  279.24     1 1006.35  3.6853 0.055176 .
Region       189.80  189.80     1  903.54  2.5049 0.113839
COVID:Region 559.32  559.32     1 1006.14  7.3816 0.006702 **
---
Signif. codes:  0 '***' 0.001 '**' 0.01 '*' 0.05 '.' 0.1 ' ' 1


$`simple contrasts for COVID`
Region = Gut:
 contrast estimate    SE   df t.ratio p.value
 No - Yes    3.707 1.384 1005   2.680  0.0075

Region = Oral/URT:
 contrast estimate    SE   df t.ratio p.value
 No - Yes   -0.638 0.803 1010  -0.794  0.4272

Degrees-of-freedom method: kenward-roger

$`simple contrasts for Region`
COVID = No:
 contrast         estimate   SE  df t.ratio p.value
 Gut - (Oral/URT)   4.2480 1.62 980   2.616  0.0090

COVID = Yes:
 contrast         estimate   SE  df t.ratio p.value
 Gut - (Oral/URT)  -0.0969 1.47 935  -0.066  0.9473

Degrees-of-freedom method: kenward-roger
```

3.1.4 Community distances

.

3.1.4.1 Model fit quality .

3.1.4.2 Model fit summary .

```
Linear mixed model fit by REML. t-tests use Satterthwaite's method [
lmerModLmerTest]
Formula: "Dist ~ COVID * Region + (1|Study.Accession)"
   Data: df.dist
```

```
REML criterion at convergence: -60329.2

Scaled residuals:
    Min      1Q  Median      3Q     Max 
-6.0124 -0.2383  0.3913  0.6157  2.9741 

Random effects:
 Groups          Name        Variance Std.Dev.
 Study.Accession (Intercept) 0.01996  0.1413  
 Residual                    0.02229  0.1493  
Number of obs: 62546, groups:  Study.Accession, 8

Fixed effects:
                          Estimate Std. Error         df t value Pr(>|t|)    
(Intercept)              8.151e-01  5.038e-02  7.033e+00  16.179 8.01e-07 ***
COVIDYes                 6.592e-02  5.515e-03  6.254e+04  11.954  < 2e-16 ***
RegionOral/URT          -1.167e-01  6.539e-03  6.244e+04 -17.847  < 2e-16 ***
COVIDYes:RegionOral/URT -2.268e-02  6.530e-03  6.254e+04  -3.473 0.000516 ***
---
Signif. codes:  0 '***' 0.001 '**' 0.01 '*' 0.05 '.' 0.1 ' ' 1

Correlation of Fixed Effects:
            (Intr) COVIDYs RO/URT
COVIDYes    -0.077               
RegnOrl/URT -0.100  0.600        
COVIDY:RO/U  0.059 -0.844  -0.675

Type III Analysis of Variance Table with Satterthwaite's method
             Sum Sq Mean Sq NumDF DenDF F value    Pr(>F)    
COVID        6.2289  6.2289     1 62541  279.48 < 2.2e-16 ***
Region      14.8469 14.8469     1 62082  666.17 < 2.2e-16 ***
COVID:Region  0.2688  0.2688     1 62540   12.06 0.0005156 ***
---
Signif. codes:  0 '***' 0.001 '**' 0.01 '*' 0.05 '.' 0.1 ' ' 1
```

Gut microbiome

.

```
Linear mixed model fit by REML. t-tests use Satterthwaite's method [
lmerModLmerTest]
Formula: "Dist ~ COVID + (1|Study.Accession)"
   Data: filter(df.dist, Region == "Gut")

REML criterion at convergence: -6783.1

Scaled residuals:
    Min      1Q  Median      3Q     Max 
-7.9945 -0.3002  0.0160  0.7454  2.0119 

Random effects:
 Groups          Name        Variance Std.Dev.
 Study.Accession (Intercept) 0.016825 0.12971 
```

```
 Residual                    0.008171 0.09039
Number of obs: 3458, groups:  Study.Accession, 2

Fixed effects:
             Estimate Std. Error        df t value Pr(>|t|)
(Intercept) 8.409e-01  9.176e-02 1.001e+00   9.164    0.069 .
COVIDYes    6.593e-02  3.339e-03 3.455e+03  19.744   <2e-16 ***
---
Signif. codes:  0 '***' 0.001 '**' 0.01 '*' 0.05 '.' 0.1 ' ' 1

Correlation of Fixed Effects:
         (Intr)
COVIDYes -0.025
```

Oral/URL microbiome

.

```
Linear mixed model fit by REML. t-tests use Satterthwaite's method [
lmerModLmerTest]
Formula: "Dist ~ COVID + (1|Study.Accession)"
   Data: filter(df.dist, Region != "Gut")

REML criterion at convergence: -54863.7

Scaled residuals:
    Min      1Q  Median      3Q     Max
-5.9040 -0.2406  0.4007  0.6079  2.9228

Random effects:
 Groups          Name        Variance Std.Dev.
 Study.Accession (Intercept) 0.02069  0.1438
 Residual                    0.02311  0.1520
Number of obs: 59088, groups:  Study.Accession, 7

Fixed effects:
             Estimate Std. Error        df t value Pr(>|t|)
(Intercept) 6.816e-01  5.459e-02 5.937e+00   12.49 1.74e-05 ***
COVIDYes    4.326e-02  3.562e-03 5.908e+04   12.15  < 2e-16 ***
---
Signif. codes:  0 '***' 0.001 '**' 0.01 '*' 0.05 '.' 0.1 ' ' 1

Correlation of Fixed Effects:
         (Intr)
COVIDYes -0.052
```

3.2 Effect of COVID per sampling method

.

3.2.1 Hill q=0

.

3.2.1.1 Model fit quality .

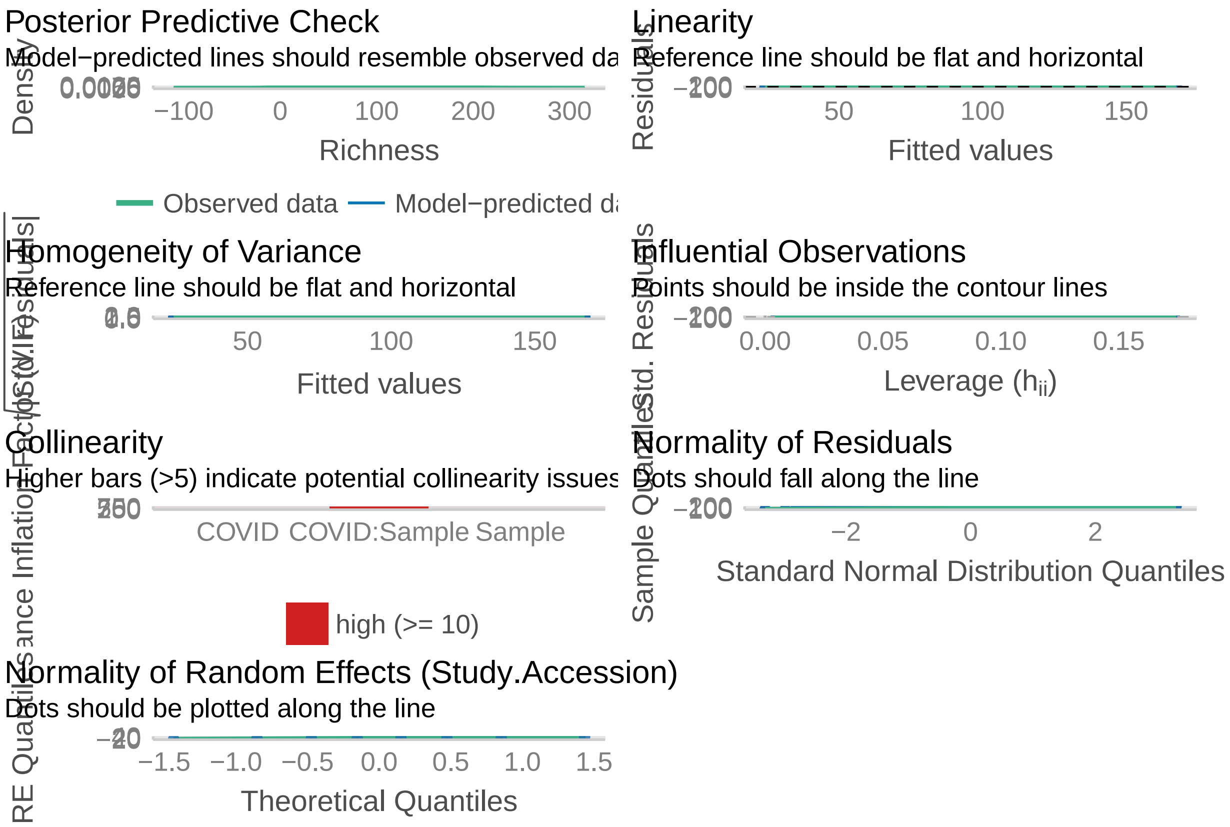

3.2.1.2 Model fit summary .

```
Linear mixed model fit by REML. t-tests use Satterthwaite's method [
lmerModLmerTest]
Formula: "Richness ~ COVID*Sample + (1|Study.Accession)"
   Data: df.1

REML criterion at convergence: 10251.3

Scaled residuals:
    Min      1Q  Median      3Q     Max
-3.7359 -0.6156 -0.1740  0.5057  4.7754

Random effects:
 Groups          Name        Variance Std.Dev.
 Study.Accession (Intercept)  327.3   18.09
 Residual                    1500.5   38.74
Number of obs: 1016, groups:  Study.Accession, 8

Fixed effects:
                                   Estimate Std. Error       df t value
(Intercept)                          49.935     18.531   74.256   2.695
COVIDYes                              5.012     16.881  999.573   0.297
```

```
SampleFaeces                        106.152     23.017   20.667   4.612
SampleNasal swab                    -21.232     26.511   35.564  -0.801
SampleNasopharyngeal swab             6.725     16.613 1001.380   0.405
SampleOropharyngeal swab             11.227     17.192  999.573   0.653
SampleThroat swab                   122.061     23.500   22.354   5.194
COVIDYes:SampleFaeces               -27.456     17.969  999.577  -1.528
COVIDYes:SampleNasal swab            10.629     23.343  968.187   0.455
COVIDYes:SampleNasopharyngeal swab   -1.219     17.661 1000.403  -0.069
COVIDYes:SampleOropharyngeal swab     7.475     18.362  999.573   0.407
COVIDYes:SampleThroat swab          -23.817     18.751  999.573  -1.270
                                   Pr(>|t|)
(Intercept)                        0.008707 **
COVIDYes                           0.766625
SampleFaeces                       0.000156 ***
SampleNasal swab                   0.428522
SampleNasopharyngeal swab          0.685705
SampleOropharyngeal swab           0.513867
SampleThroat swab                  3.14e-05 ***
COVIDYes:SampleFaeces              0.126841
COVIDYes:SampleNasal swab          0.648974
COVIDYes:SampleNasopharyngeal swab 0.944968
COVIDYes:SampleOropharyngeal swab  0.684008
COVIDYes:SampleThroat swab         0.204303
---
Signif. codes:  0 '***' 0.001 '**' 0.01 '*' 0.05 '.' 0.1 ' ' 1

Correlation of Fixed Effects:
                 (Intr) COVIDYes SmplFc SmplNsls SmplNsps SmplOs SmplTs COVIDY:
COVIDYes         -0.799
SampleFaecs      -0.805  0.644
SamplNslswb      -0.699  0.559    0.563
SmplNsphrys      -0.837  0.892    0.674  0.585
SmplOrphrys      -0.785  0.862    0.632  0.549    0.876
SmplThrtswb      -0.789  0.630    0.937  0.551    0.660    0.619
COVIDYs:SmF       0.751 -0.939   -0.660 -0.525   -0.838   -0.810 -0.591
COVIDYs:SmplNsls  0.578 -0.723   -0.465 -0.739   -0.645   -0.623 -0.456  0.679
COVIDYs:SmplNsps  0.776 -0.956   -0.624 -0.542   -0.932   -0.824 -0.612  0.898
COVIDYs:SOs       0.735 -0.919   -0.592 -0.514   -0.820   -0.936 -0.580  0.864
COVIDYs:STs       0.720 -0.900   -0.579 -0.503   -0.803   -0.776 -0.651  0.846
                 COVIDYs:SmplNsls COVIDYs:SmplNsps COVIDY:SOs
COVIDYes
SampleFaecs
SamplNslswb
SmplNsphrys
SmplOrphrys
SmplThrtswb
COVIDYs:SmF
COVIDYs:SmplNsls
COVIDYs:SmplNsps  0.691
COVIDYs:SOs       0.665            0.879
COVIDYs:STs       0.651            0.861            0.828

Type III Analysis of Variance Table with Satterthwaite's method
             Sum Sq Mean Sq NumDF  DenDF F value    Pr(>F)
```

```
COVID            38    38.4     1 987.34  0.0256 0.8729010
Sample        83211 16642.2     5  17.59 11.0910 6.055e-05 ***
COVID:Sample  32261  6452.2     5 966.39  4.3000 0.0007117 ***
---
Signif. codes:  0 '***' 0.001 '**' 0.01 '*' 0.05 '.' 0.1 ' ' 1

$`simple contrasts for COVID`
Sample = Endotracheal aspirate:
 contrast estimate    SE   df t.ratio p.value
 No - Yes    -5.01 16.88  999  -0.297  0.7666

Sample = Faeces:
 contrast estimate    SE   df t.ratio p.value
 No - Yes    22.44  6.16  999   3.645  0.0003

Sample = Nasal swab:
 contrast estimate    SE   df t.ratio p.value
 No - Yes   -15.64 16.35  800  -0.956  0.3392

Sample = Nasopharyngeal swab:
 contrast estimate    SE   df t.ratio p.value
 No - Yes    -3.79  5.20 1004  -0.729  0.4661

Sample = Oropharyngeal swab:
 contrast estimate    SE   df t.ratio p.value
 No - Yes   -12.49  7.22  999  -1.729  0.0841

Sample = Throat swab:
 contrast estimate    SE   df t.ratio p.value
 No - Yes    18.81  8.16  999   2.304  0.0214

Degrees-of-freedom method: kenward-roger

$`simple contrasts for Sample`
COVID = No:
 contrast                                    estimate    SE      df t.ratio
 Endotracheal aspirate - Faeces               -106.15 23.03   18.43  -4.609
 Endotracheal aspirate - Nasal swab             21.23 26.55   31.77   0.800
 Endotracheal aspirate - Nasopharyngeal swab    -6.73 16.62 1001.06  -0.405
 Endotracheal aspirate - Oropharyngeal swab    -11.23 17.19  999.03  -0.653
 Endotracheal aspirate - Throat swab          -122.06 23.52   19.94  -5.189
 Faeces - Nasal swab                           127.38 23.39   11.00   5.446
 Faeces - Nasopharyngeal swab                   99.43 17.05    5.60   5.831
 Faeces - Oropharyngeal swab                    94.92 18.05    6.97   5.258
 Faeces - Throat swab                          -15.91  8.30 1002.30  -1.916
 Nasal swab - Nasopharyngeal swab              -27.96 21.57   14.03  -1.296
 Nasal swab - Oropharyngeal swab               -32.46 22.37   16.10  -1.451
 Nasal swab - Throat swab                     -143.29 23.88   11.89  -6.002
 Nasopharyngeal swab - Oropharyngeal swab       -4.50  8.46 1003.94  -0.532
 Nasopharyngeal swab - Throat swab            -115.34 17.71    6.48  -6.512
 Oropharyngeal swab - Throat swab             -110.83 18.68    7.94  -5.935
 p.value
  0.0024
  0.9655
```

```
  0.9986
  0.9868
  0.0006
  0.0021
  0.0103
  0.0096
  0.3928
  0.7824
  0.6974
  0.0007
  0.9949
  0.0037
  0.0032

COVID = Yes:
 contrast                                   estimate    SE      df t.ratio
 Endotracheal aspirate - Faeces               -78.70 17.54    6.22  -4.486
 Endotracheal aspirate - Nasal swab            10.60 18.34    7.13   0.578
 Endotracheal aspirate - Nasopharyngeal swab   -5.51  6.43 1001.73  -0.856
 Endotracheal aspirate - Oropharyngeal swab   -18.70  6.45  999.03  -2.900
 Endotracheal aspirate - Throat swab          -98.24 18.21    7.17  -5.395
 Faeces - Nasal swab                           89.30 19.65    5.39   4.543
 Faeces - Nasopharyngeal swab                  73.19 16.48    4.85   4.442
 Faeces - Oropharyngeal swab                   59.99 16.72    5.13   3.588
 Faeces - Throat swab                         -19.55  7.35 1000.25  -2.659
 Nasal swab - Nasopharyngeal swab             -16.11 17.33    5.69  -0.930
 Nasal swab - Oropharyngeal swab              -29.31 17.56    5.98  -1.669
 Nasal swab - Throat swab                    -108.85 20.25    6.06  -5.374
 Nasopharyngeal swab - Oropharyngeal swab     -13.20  3.63 1004.00  -3.640
 Nasopharyngeal swab - Throat swab            -92.74 17.19    5.71  -5.396
 Oropharyngeal swab - Throat swab             -79.54 17.42    6.00  -4.566
 p.value
  0.0278
  0.9895
  0.9566
  0.0441
  0.0077
  0.0342
  0.0450
  0.0911
  0.0845
  0.9240
  0.5909
  0.0124
  0.0039
  0.0141
  0.0273

Degrees-of-freedom method: kenward-roger
P value adjustment: tukey method for comparing a family of 6 estimates
```

3.2.2 Hill q=1

.

3.2.2.1 Model fit quality .

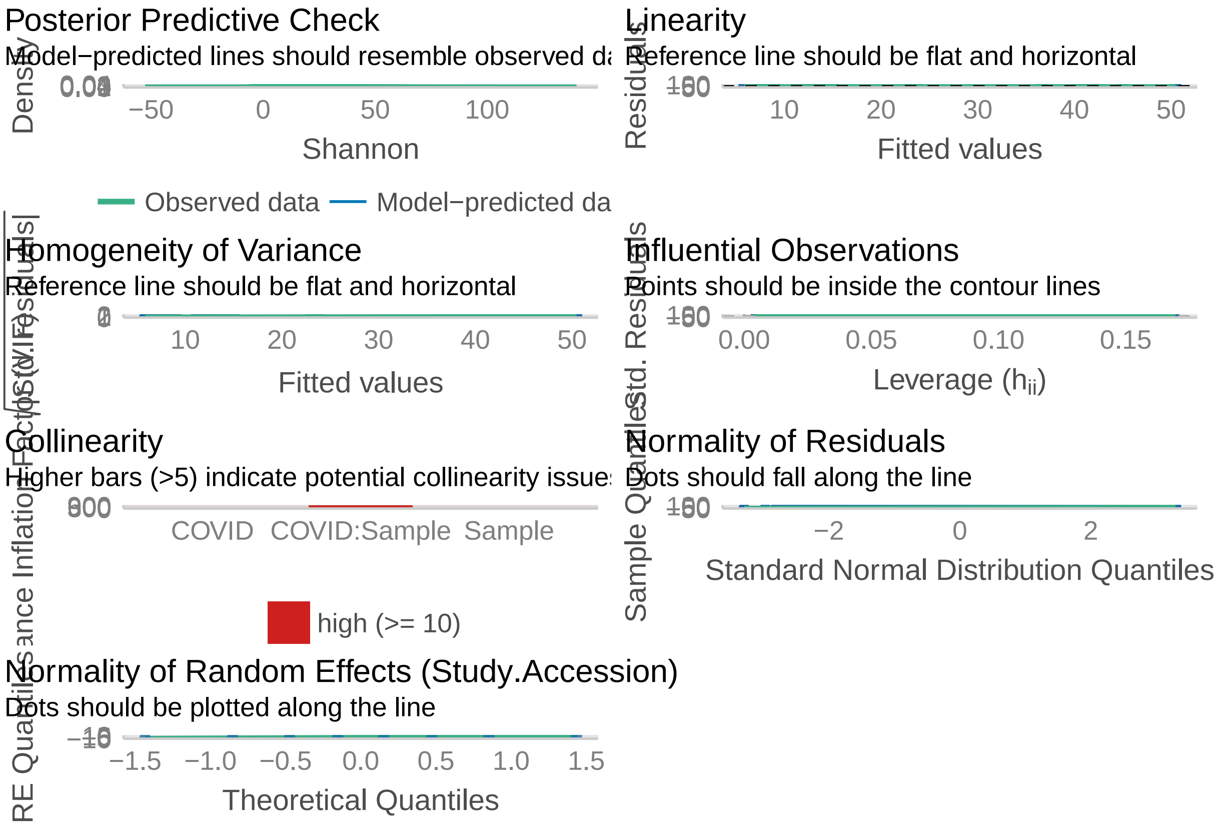

3.2.2.2 Model fit summary .

```
Linear mixed model fit by REML. t-tests use Satterthwaite's method [
lmerModLmerTest]
Formula: "Shannon ~ COVID*Sample + (1|Study.Accession)"
   Data: df.1

REML criterion at convergence: 8434.7

Scaled residuals:
    Min      1Q  Median      3Q     Max
-3.0791 -0.4450 -0.2117  0.3018  8.1264

Random effects:
 Groups          Name        Variance Std.Dev.
 Study.Accession (Intercept)  35.17    5.931
 Residual                    246.17   15.690
Number of obs: 1016, groups:  Study.Accession, 8
```

```
Fixed effects:
                                   Estimate Std. Error        df t value
(Intercept)                         13.2837     7.1860  124.4007   1.849
COVIDYes                             0.3283     6.8376  999.6270   0.048
SampleFaeces                        34.3920     8.5414   30.5707   4.027
SampleNasal swab                    -3.6670    10.0605   56.3405  -0.364
SampleNasopharyngeal swab            1.2053     6.7272 1002.0828   0.179
SampleOropharyngeal swab             2.4686     6.9633  999.6270   0.355
SampleThroat swab                   29.6335     8.7524   33.3733   3.386
COVIDYes:SampleFaeces               -8.5268     7.2782  999.6339  -1.172
COVIDYes:SampleNasal swab           -1.4214     9.4157  933.3947  -0.151
COVIDYes:SampleNasopharyngeal swab   0.4216     7.1526 1000.7445   0.059
COVIDYes:SampleOropharyngeal swab    2.9192     7.4372  999.6270   0.393
COVIDYes:SampleThroat swab           0.3942     7.5948  999.6270   0.052
                                   Pr(>|t|)
(Intercept)                        0.066898 .
COVIDYes                           0.961715
SampleFaeces                       0.000346 ***
SampleNasal swab                   0.716855
SampleNasopharyngeal swab          0.857840
SampleOropharyngeal swab           0.723029
SampleThroat swab                  0.001831 **
COVIDYes:SampleFaeces              0.241653
COVIDYes:SampleNasal swab          0.880039
COVIDYes:SampleNasopharyngeal swab 0.953006
COVIDYes:SampleOropharyngeal swab  0.694763
COVIDYes:SampleThroat swab         0.958613
---
Signif. codes:  0 ’***’ 0.001 ’**’ 0.01 ’*’ 0.05 ’.’ 0.1 ’ ’ 1

Correlation of Fixed Effects:
                 (Intr) COVIDYes SmplFc SmplNsls SmplNsps SmplOs SmplTs COVIDY:
COVIDYes         -0.835
SampleFaecs      -0.841  0.703
SamplNslswb      -0.714  0.596    0.601
SmplNsphrys      -0.873  0.892    0.735  0.624
SmplOrphrys      -0.820  0.862    0.690  0.586    0.876
SmplThrtswb      -0.821  0.686    0.925  0.586    0.717    0.673
COVIDYs:SmF       0.784 -0.939   -0.720 -0.560   -0.838   -0.810 -0.643
COVIDYs:SmplNsls  0.606 -0.726   -0.510 -0.789   -0.648   -0.626 -0.498  0.682
COVIDYs:SmplNsps  0.810 -0.956   -0.681 -0.578   -0.932   -0.824 -0.665  0.898
COVIDYs:SOs       0.768 -0.919   -0.646 -0.548   -0.820   -0.936 -0.630  0.864
COVIDYs:STs       0.752 -0.900   -0.632 -0.537   -0.803   -0.776 -0.708  0.846
                 COVIDYs:SmplNsls COVIDYs:SmplNsps COVIDY:SOs
COVIDYes
SampleFaecs
SamplNslswb
SmplNsphrys
SmplOrphrys
SmplThrtswb
COVIDYs:SmF
COVIDYs:SmplNsls
COVIDYs:SmplNsps  0.694
COVIDYs:SOs       0.668            0.879
```

```
COVIDYs:STs       0.654            0.861            0.828

Type III Analysis of Variance Table with Satterthwaite's method
             Sum Sq Mean Sq NumDF  DenDF F value    Pr(>F)
COVID          37.3   37.32     1 968.13  0.1516 0.6970891
Sample       9289.4 1857.88     5  19.15  7.5472 0.0004614 ***
COVID:Sample 2814.9  562.99     5 936.35  2.2870 0.0442775 *
---
Signif. codes:  0 '***' 0.001 '**' 0.01 '*' 0.05 '.' 0.1 ' ' 1

$`simple contrasts for COVID`
Sample = Endotracheal aspirate:
 contrast estimate   SE   df t.ratio p.value
 No - Yes   -0.328 6.84  999  -0.048  0.9617

Sample = Faeces:
 contrast estimate   SE   df t.ratio p.value
 No - Yes    8.198 2.49  999   3.288  0.0010

Sample = Nasal swab:
 contrast estimate   SE   df t.ratio p.value
 No - Yes    1.093 6.59  675   0.166  0.8683

Sample = Nasopharyngeal swab:
 contrast estimate   SE   df t.ratio p.value
 No - Yes   -0.750 2.11 1002  -0.356  0.7218

Sample = Oropharyngeal swab:
 contrast estimate   SE   df t.ratio p.value
 No - Yes   -3.247 2.93  999  -1.110  0.2672

Sample = Throat swab:
 contrast estimate   SE   df t.ratio p.value
 No - Yes   -0.723 3.31  999  -0.219  0.8270

Degrees-of-freedom method: kenward-roger

$`simple contrasts for Sample`
COVID = No:
 contrast                                    estimate    SE      df t.ratio
 Endotracheal aspirate - Faeces                -34.39  8.55   27.11  -4.022
 Endotracheal aspirate - Nasal swab              3.67 10.08   50.12   0.364
 Endotracheal aspirate - Nasopharyngeal swab    -1.21  6.73 1001.83  -0.179
 Endotracheal aspirate - Oropharyngeal swab     -2.47  6.96  999.05  -0.355
 Endotracheal aspirate - Throat swab           -29.63  8.77   29.61  -3.380
 Faeces - Nasal swab                            38.06  8.44   14.42   4.512
 Faeces - Nasopharyngeal swab                   33.19  5.81    5.93   5.707
 Faeces - Oropharyngeal swab                    31.92  6.29    7.97   5.076
 Faeces - Throat swab                            4.76  3.36  994.08   1.415
 Nasal swab - Nasopharyngeal swab               -4.87  7.90   19.41  -0.617
 Nasal swab - Oropharyngeal swab                -6.14  8.25   22.78  -0.744
 Nasal swab - Throat swab                      -33.30  8.66   15.83  -3.847
 Nasopharyngeal swab - Oropharyngeal swab       -1.26  3.43 1003.52  -0.369
 Nasopharyngeal swab - Throat swab             -28.43  6.13    7.22  -4.638
```

```
 Oropharyngeal swab - Throat swab             -27.16  6.58    9.44  -4.127
 p.value
  0.0050
  0.9991
  1.0000
  0.9993
  0.0228
  0.0049
  0.0097
  0.0084
  0.7180
  0.9884
  0.9741
  0.0150
  0.9991
  0.0175
  0.0205

COVID = Yes:
 contrast                                    estimate    SE      df t.ratio
 Endotracheal aspirate - Faeces                -25.87  6.05    6.81  -4.277
 Endotracheal aspirate - Nasal swab              5.09  6.41    8.01   0.793
 Endotracheal aspirate - Nasopharyngeal swab    -1.63  2.60 1002.78  -0.625
 Endotracheal aspirate - Oropharyngeal swab     -5.39  2.61  999.05  -2.063
 Endotracheal aspirate - Throat swab           -30.03  6.36    8.24  -4.718
 Faeces - Nasal swab                            30.95  6.69    5.52   4.629
 Faeces - Nasopharyngeal swab                   24.24  5.53    4.81   4.380
 Faeces - Oropharyngeal swab                    20.48  5.65    5.19   3.623
 Faeces - Throat swab                           -4.16  2.98  987.18  -1.398
 Nasal swab - Nasopharyngeal swab               -6.72  5.93    5.87  -1.132
 Nasal swab - Oropharyngeal swab               -10.48  6.04    6.28  -1.734
 Nasal swab - Throat swab                      -35.12  6.97    6.47  -5.036
 Nasopharyngeal swab - Oropharyngeal swab       -3.76  1.47 1002.27  -2.561
 Nasopharyngeal swab - Throat swab             -28.40  5.88    6.04  -4.832
 Oropharyngeal swab - Throat swab              -24.64  5.99    6.46  -4.114
 p.value
  0.0295
  0.9610
  0.9892
  0.3080
  0.0120
  0.0303
  0.0482
  0.0868
  0.7283
  0.8524
  0.5568
  0.0146
  0.1080
  0.0208
  0.0387

Degrees-of-freedom method: kenward-roger
P value adjustment: tukey method for comparing a family of 6 estimates
```

3.2.3 Hill q=2

.

3.2.3.1 Model fit quality .

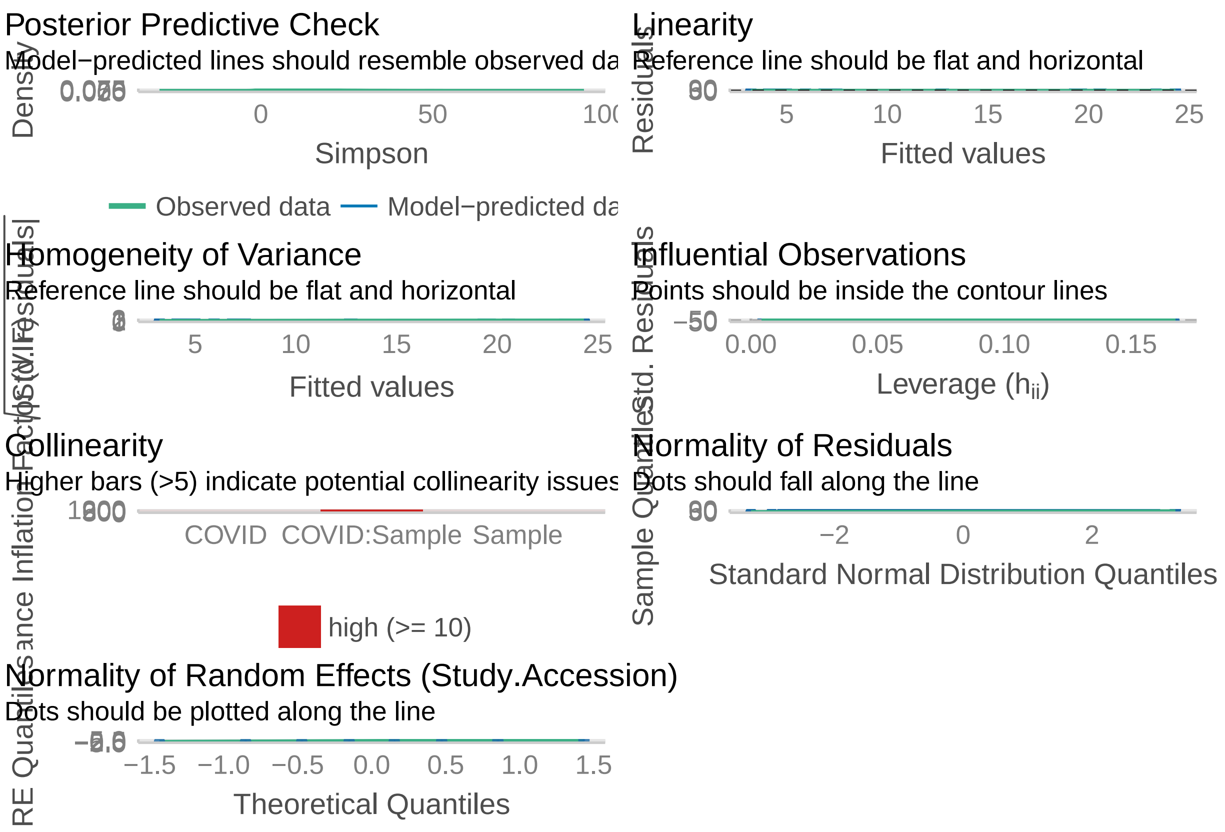

3.2.3.2 Model fit summary .

```
Linear mixed model fit by REML. t-tests use Satterthwaite's method [
lmerModLmerTest]
Formula: "Simpson ~ COVID*Sample + (1|Study.Accession)"
   Data: df.1

REML criterion at convergence: 7246.6

Scaled residuals:
    Min      1Q  Median      3Q     Max
-2.6228 -0.4290 -0.2135  0.2669 10.0068

Random effects:
 Groups          Name        Variance Std.Dev.
 Study.Accession (Intercept)  9.161   3.027
 Residual                    75.444   8.686
Number of obs: 1016, groups:  Study.Accession, 8
```

```
Fixed effects:
                                   Estimate Std. Error        df t value
(Intercept)                          7.5538     3.9258  149.7694   1.924
COVIDYes                            -0.4416     3.7853  999.6242  -0.117
SampleFaeces                        16.2779     4.5974   35.6902   3.541
SampleNasal swab                    -1.4071     5.4570   67.0723  -0.258
SampleNasopharyngeal swab            0.3280     3.7237 1002.3719   0.088
SampleOropharyngeal swab             0.4503     3.8549  999.6242   0.117
SampleThroat swab                   12.2262     4.7168   39.0310   2.592
COVIDYes:SampleFaeces               -3.2594     4.0292  999.6322  -0.809
COVIDYes:SampleNasal swab           -1.1200     5.2030  916.3093  -0.215
COVIDYes:SampleNasopharyngeal swab   0.5492     3.9595 1000.8711   0.139
COVIDYes:SampleOropharyngeal swab    2.5379     4.1172  999.6242   0.616
COVIDYes:SampleThroat swab           1.6711     4.2044  999.6242   0.397
                                  Pr(>|t|)
(Intercept)                        0.05623 .
COVIDYes                           0.90716
SampleFaeces                       0.00113 **
SampleNasal swab                   0.79731
SampleNasopharyngeal swab          0.92982
SampleOropharyngeal swab           0.90703
SampleThroat swab                  0.01336 *
COVIDYes:SampleFaeces              0.41873
COVIDYes:SampleNasal swab          0.82961
COVIDYes:SampleNasopharyngeal swab  0.88971
COVIDYes:SampleOropharyngeal swab  0.53776
COVIDYes:SampleThroat swab         0.69112
---
Signif. codes:  0 ’***’ 0.001 ’**’ 0.01 ’*’ 0.05 ’.’ 0.1 ’ ’ 1

Correlation of Fixed Effects:
                 (Intr) COVIDYes SmplFc SmplNsls SmplNsps SmplOs SmplTs COVIDY:
COVIDYes         -0.846
SampleFaecs      -0.854  0.723
SamplNslswb      -0.719  0.609    0.614
SmplNsphrys      -0.885  0.892    0.755  0.636
SmplOrphrys      -0.831  0.862    0.709  0.598    0.876
SmplThrtswb      -0.832  0.704    0.921  0.599    0.736    0.692
COVIDYs:SmF       0.795 -0.939   -0.741 -0.572   -0.838   -0.810 -0.661
COVIDYs:SmplNsls  0.616 -0.728   -0.526 -0.805   -0.649   -0.627 -0.512  0.683
COVIDYs:SmplNsps  0.820 -0.956   -0.700 -0.590   -0.932   -0.824 -0.683  0.898
COVIDYs:SOs       0.778 -0.919   -0.664 -0.560   -0.820   -0.936 -0.647  0.864
COVIDYs:STs       0.762 -0.900   -0.651 -0.548   -0.803   -0.776 -0.727  0.846
                 COVIDYs:SmplNsls COVIDYs:SmplNsps COVIDY:SOs
COVIDYes
SampleFaecs
SamplNslswb
SmplNsphrys
SmplOrphrys
SmplThrtswb
COVIDYs:SmF
COVIDYs:SmplNsls
COVIDYs:SmplNsps  0.696
COVIDYs:SOs       0.669            0.879
```

```
COVIDYs:STs       0.655            0.861            0.828

Type III Analysis of Variance Table with Satterthwaite's method
              Sum Sq Mean Sq NumDF  DenDF F value    Pr(>F)
COVID          10.72   10.72     1 958.31  0.1420 0.7063387
Sample       2470.50  494.10     5  19.45  6.5493 0.0009975 ***
COVID:Sample  692.82  138.56     5 921.40  1.8366 0.1031292
---
Signif. codes:  0 '***' 0.001 '**' 0.01 '*' 0.05 '.' 0.1 ' ' 1

$`simple contrasts for COVID`
Sample = Endotracheal aspirate:
 contrast estimate   SE   df t.ratio p.value
 No - Yes    0.442 3.79  999   0.117  0.9072

Sample = Faeces:
 contrast estimate   SE   df t.ratio p.value
 No - Yes    3.701 1.38  999   2.681  0.0075

Sample = Nasal swab:
 contrast estimate   SE   df t.ratio p.value
 No - Yes    1.562 3.64  626   0.429  0.6681

Sample = Nasopharyngeal swab:
 contrast estimate   SE   df t.ratio p.value
 No - Yes   -0.108 1.17 1001  -0.092  0.9264

Sample = Oropharyngeal swab:
 contrast estimate   SE   df t.ratio p.value
 No - Yes   -2.096 1.62  999  -1.294  0.1958

Sample = Throat swab:
 contrast estimate   SE   df t.ratio p.value
 No - Yes   -1.229 1.83  999  -0.672  0.5018

Degrees-of-freedom method: kenward-roger

$`simple contrasts for Sample`
COVID = No:
 contrast                                    estimate    SE      df t.ratio
 Endotracheal aspirate - Faeces               -16.278 4.604   31.79  -3.536
 Endotracheal aspirate - Nasal swab             1.407 5.471   59.96   0.257
 Endotracheal aspirate - Nasopharyngeal swab   -0.328 3.727 1002.17  -0.088
 Endotracheal aspirate - Oropharyngeal swab    -0.450 3.855  999.07  -0.117
 Endotracheal aspirate - Throat swab          -12.226 4.727   34.78  -2.586
 Faeces - Nasal swab                           17.685 4.492   16.20   3.937
 Faeces - Nasopharyngeal swab                  15.950 3.025    6.10   5.273
 Faeces - Oropharyngeal swab                   15.828 3.303    8.47   4.792
 Faeces - Throat swab                           4.052 1.862  987.41   2.176
 Nasal swab - Nasopharyngeal swab              -1.735 4.228   22.23  -0.410
 Nasal swab - Oropharyngeal swab               -1.857 4.432   26.28  -0.419
 Nasal swab - Throat swab                     -13.633 4.619   17.88  -2.952
 Nasopharyngeal swab - Oropharyngeal swab      -0.122 1.898 1002.69  -0.064
 Nasopharyngeal swab - Throat swab            -11.898 3.209    7.59  -3.707
```

```
 Oropharyngeal swab - Throat swab            -11.776 3.473   10.18  -3.391
 p.value
  0.0147
  0.9998
  1.0000
  1.0000
  0.1278
  0.0122
  0.0133
  0.0103
  0.2499
  0.9983
  0.9982
  0.0779
  1.0000
  0.0498
  0.0557

COVID = Yes:
 contrast                                   estimate    SE      df t.ratio
 Endotracheal aspirate - Faeces              -13.018 3.161    7.11  -4.118
 Endotracheal aspirate - Nasal swab            2.527 3.372    8.41   0.749
 Endotracheal aspirate - Nasopharyngeal swab   -0.877 1.442 1003.19  -0.608
 Endotracheal aspirate - Oropharyngeal swab   -2.988 1.446  999.07  -2.066
 Endotracheal aspirate - Throat swab         -13.897 3.346    8.77  -4.153
 Faeces - Nasal swab                          15.546 3.473    5.57   4.476
 Faeces - Nasopharyngeal swab                 12.141 2.859    4.79   4.247
 Faeces - Oropharyngeal swab                  10.030 2.929    5.22   3.425
 Faeces - Throat swab                         -0.879 1.648  977.19  -0.533
 Nasal swab - Nasopharyngeal swab             -3.404 3.090    5.94  -1.102
 Nasal swab - Oropharyngeal swab              -5.515 3.155    6.40  -1.748
 Nasal swab - Throat swab                    -16.424 3.642    6.65  -4.510
 Nasopharyngeal swab - Oropharyngeal swab     -2.111 0.813 1000.16  -2.596
 Nasopharyngeal swab - Throat swab           -13.020 3.062    6.19  -4.253
 Oropharyngeal swab - Throat swab            -10.909 3.127    6.67  -3.488
 p.value
  0.0330
  0.9693
  0.9904
  0.3059
  0.0223
  0.0343
  0.0544
  0.1050
  0.9948
  0.8647
  0.5488
  0.0238
  0.0990
  0.0357
  0.0758

Degrees-of-freedom method: kenward-roger
P value adjustment: tukey method for comparing a family of 6 estimates
```

3.2.4 Community distances

.

3.2.4.1 Model fit quality .

3.2.4.2 Model fit summary .

```
Linear mixed model fit by REML. t-tests use Satterthwaite's method [
lmerModLmerTest]
Formula: "Dist ~ COVID*Sample + (1|Study.Accession)"
   Data: df.dist

REML criterion at convergence: -63719.7

Scaled residuals:
    Min      1Q  Median      3Q     Max
-6.4686 -0.2446  0.3333  0.4989  4.1748

Random effects:
 Groups          Name        Variance Std.Dev.
 Study.Accession (Intercept) 0.007711 0.08781
 Residual                    0.021098 0.14525
Number of obs: 62546, groups:  Study.Accession, 8

Fixed effects:
                                     Estimate Std. Error         df t value
(Intercept)                         8.035e-01  5.792e-02  1.465e+01  13.874
COVIDYes                            7.182e-02  3.781e-02  6.253e+04   1.899
SampleFaeces                        3.739e-02  8.503e-02  7.562e+00   0.440
SampleNasal swab                   -5.861e-01  9.294e-02  1.078e+01  -6.307
SampleNasopharyngeal swab          -9.527e-03  3.787e-02  6.253e+04  -0.252
SampleOropharyngeal swab            3.667e-02  3.803e-02  6.253e+04   0.964
SampleThroat swab                  -1.161e-01  8.508e-02  7.582e+00  -1.365
COVIDYes:SampleFaeces              -5.903e-03  3.819e-02  6.253e+04  -0.155
COVIDYes:SampleNasal swab           3.400e-01  5.686e-02  4.620e+04   5.980
COVIDYes:SampleNasopharyngeal swab -7.102e-02  3.818e-02  6.253e+04  -1.860
COVIDYes:SampleOropharyngeal swab  -4.466e-02  3.835e-02  6.253e+04  -1.165
COVIDYes:SampleThroat swab          3.421e-02  3.834e-02  6.253e+04   0.892
                                   Pr(>|t|)
(Intercept)                        7.92e-10 ***
COVIDYes                             0.0575 .
SampleFaeces                         0.6725
SampleNasal swab                   6.34e-05 ***
SampleNasopharyngeal swab            0.8014
SampleOropharyngeal swab             0.3350
SampleThroat swab                    0.2114
COVIDYes:SampleFaeces                0.8772
COVIDYes:SampleNasal swab          2.25e-09 ***
COVIDYes:SampleNasopharyngeal swab   0.0629 .
COVIDYes:SampleOropharyngeal swab    0.2442
COVIDYes:SampleThroat swab           0.3722
---
```

```
Signif. codes:  0 ’***’ 0.001 ’**’ 0.01 ’*’ 0.05 ’.’ 0.1 ’ ’ 1

Correlation of Fixed Effects:
                 (Intr) COVIDYes SmplFc SmplNsls SmplNsps SmplOs SmplTs COVIDY:
COVIDYes         -0.642
SampleFaecs      -0.681  0.437
SamplNslswb      -0.623  0.400    0.425
SmplNsphrys      -0.644  0.982    0.438  0.401
SmplOrphrys      -0.639  0.978    0.435  0.398    0.977
SmplThrtswb      -0.681  0.437    0.997  0.424    0.438    0.435
COVIDYs:SmF       0.636 -0.990   -0.439 -0.396   -0.972   -0.968 -0.433
COVIDYs:SmplNsls  0.427 -0.665   -0.291 -0.558   -0.653   -0.650 -0.291  0.658
COVIDYs:SmplNsps  0.638 -0.990   -0.435 -0.398   -0.992   -0.969 -0.434  0.980
COVIDYs:SOs       0.633 -0.986   -0.431 -0.395   -0.968   -0.992 -0.431  0.976
COVIDYs:STs       0.633 -0.986   -0.431 -0.395   -0.969   -0.965 -0.438  0.977
                 COVIDYs:SmplNsls COVIDYs:SmplNsps COVIDY:SOs
COVIDYes
SampleFaecs
SamplNslswb
SmplNsphrys
SmplOrphrys
SmplThrtswb
COVIDYs:SmF
COVIDYs:SmplNsls
COVIDYs:SmplNsps  0.659
COVIDYs:SOs       0.656            0.976
COVIDYs:STs       0.656            0.977            0.973

Type III Analysis of Variance Table with Satterthwaite’s method
              Sum Sq Mean Sq NumDF DenDF F value    Pr(>F)
COVID         2.9247  2.9247     1 47178 138.623 < 2.2e-16 ***
Sample       20.8623  4.1725     5    11 197.762 1.534e-10 ***
COVID:Sample  5.4659  1.0932     5 52206  51.813 < 2.2e-16 ***
---
Signif. codes:  0 ’***’ 0.001 ’**’ 0.01 ’*’ 0.05 ’.’ 0.1 ’ ’ 1
```

3.2.4.3 Hypothesis IV .
