# Supplementary material for: COVID-19 alters human microbiomes: a meta-analysis
Source: Front Cell Infect Microbiol. 2023 Aug 2;13:1211348. doi: 10.3389/fcimb.2023.1211348 (PMC10433767; doi:10.3389/fcimb.2023.1211348)
Supplement: Supplementary file 2 [file Table_1.docx]

**Supplementary Figures**


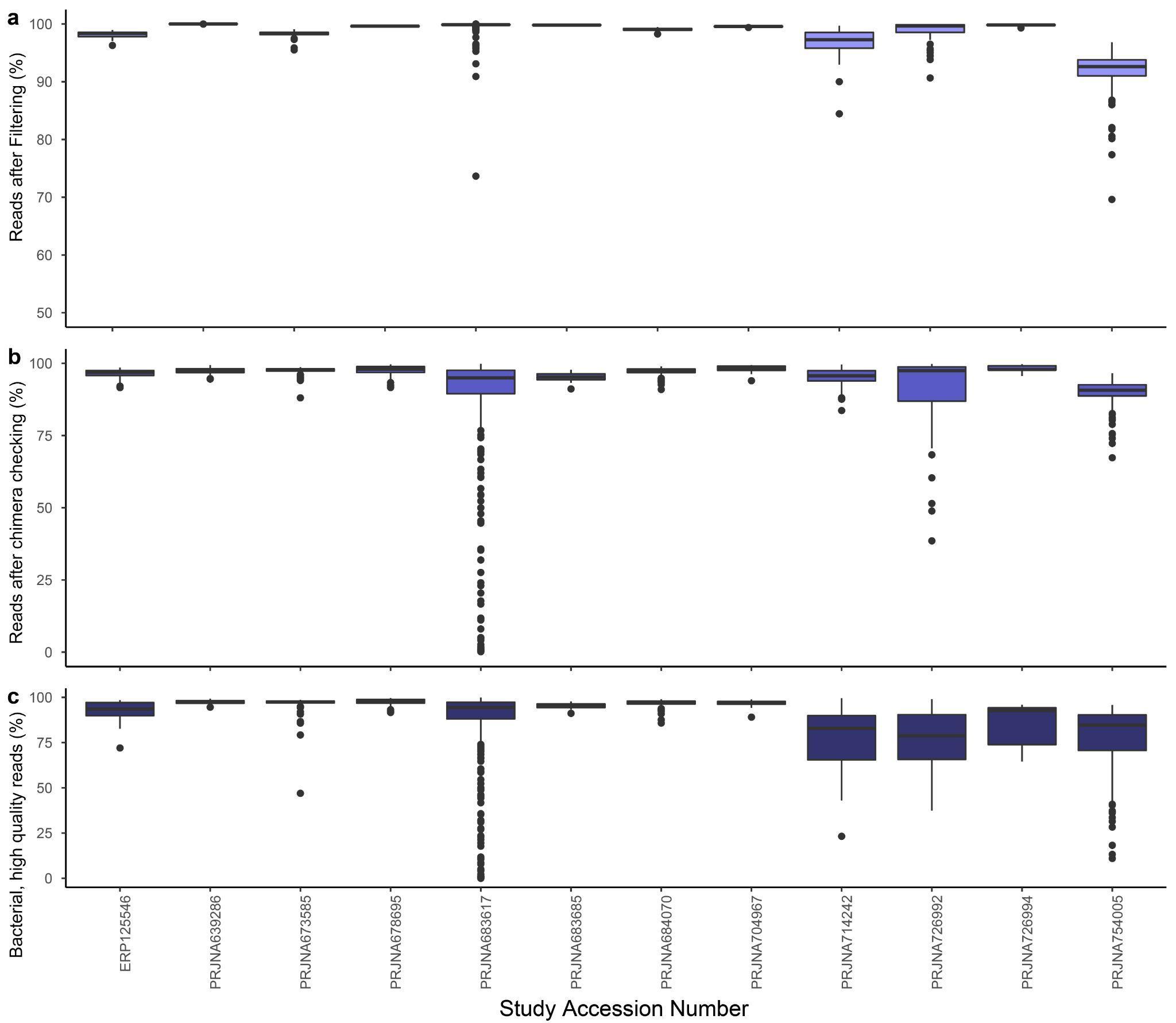


Figure S1. Proportion of reads preserved after quality filtering (a), chimera checking (b), and selection of bacterial reads (c) for each study. All data is presented as a percentage of the number of reads originally recovered from public databases Additional information for each study is found in Supplementary Table S1.


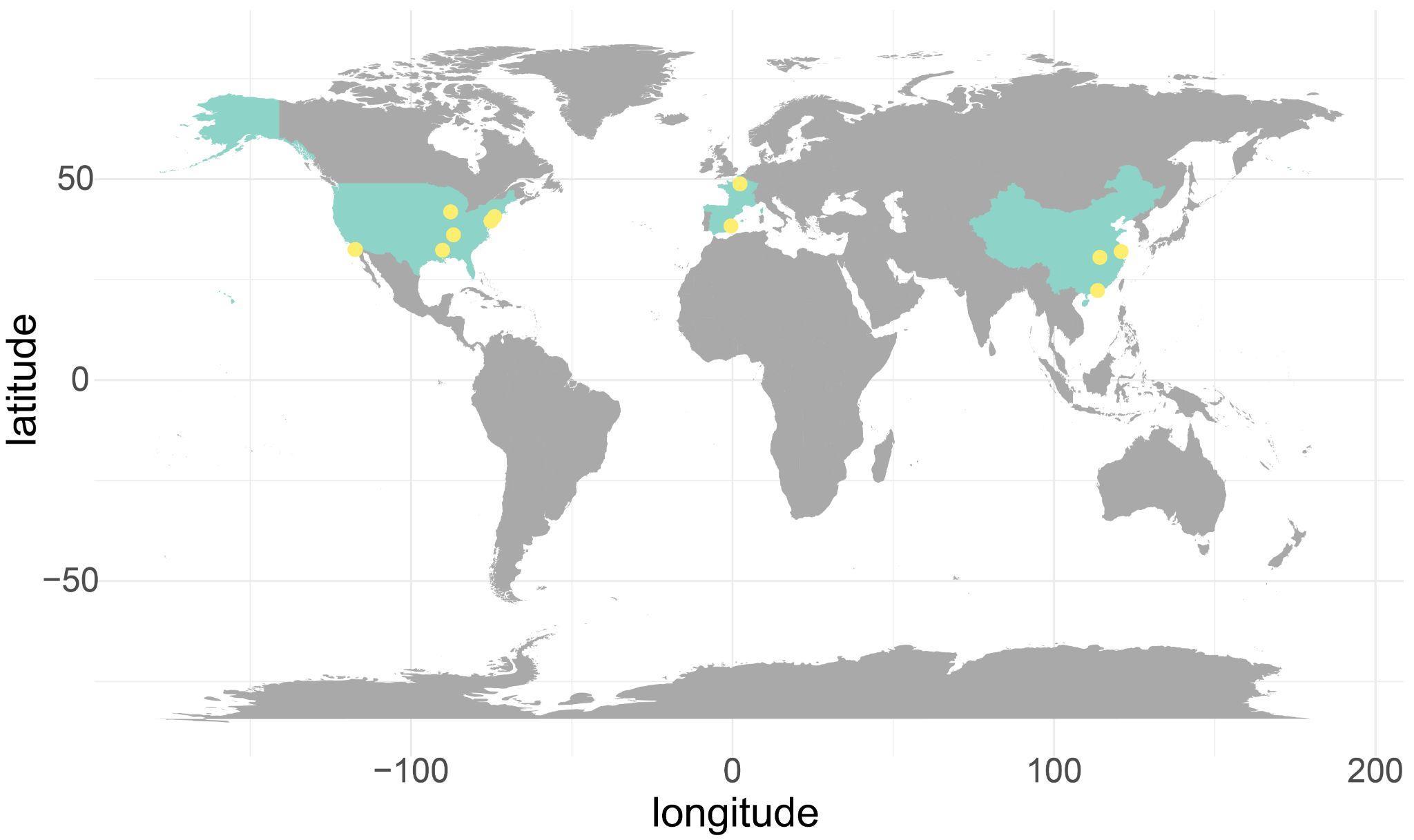


Figure S2. Distribution of individuals included in this study. Samples were collected from studies conducted in the USA (Chicago, Jackson, Nashville, New York City, Philadelphia, and San Diego), Spain (Alicante), France (Paris), and China (Guangdong, Shanghai, and Wuhan). Yellow dots indicate the locations from which samples were obtained.


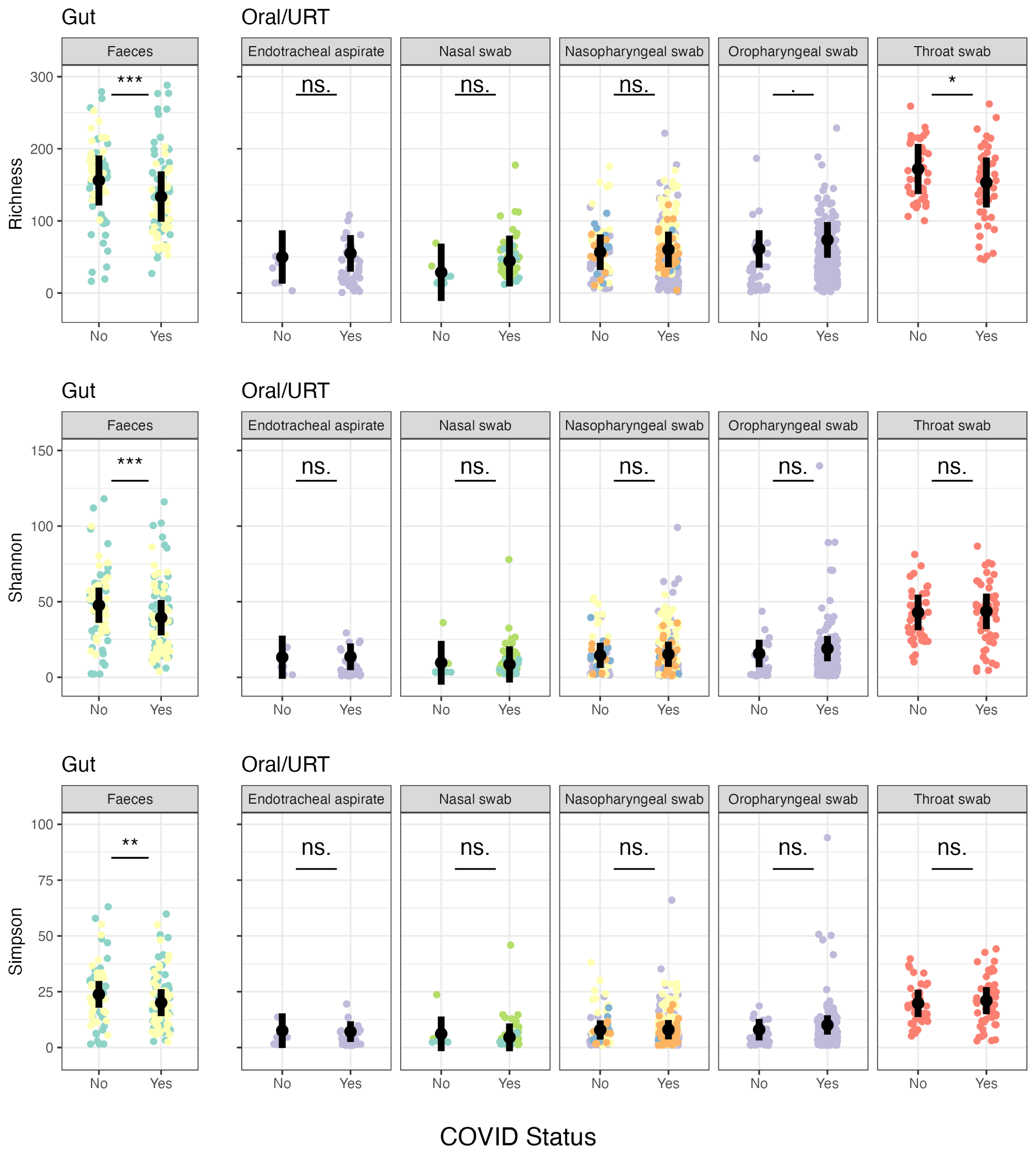


Figure S3: Effect of COVID-19 on gut and oral/URT microbiome OTU richness, Shannon and Simpson diversity according to the sampling region. Points are colored according to the study


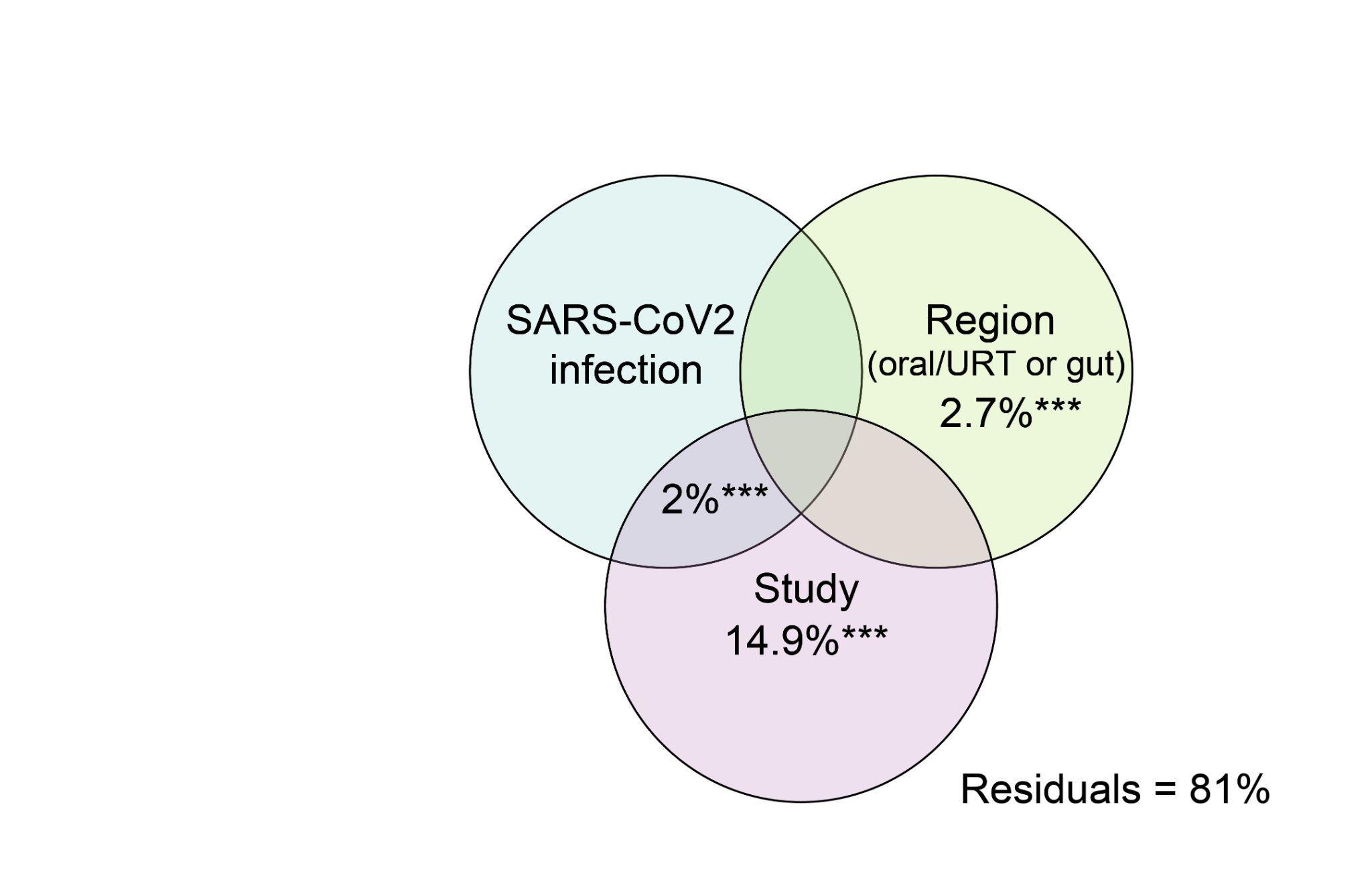


Figure S4. Variance partitioning analysis quantifying the percentage of variance in microbiome composition explained by SARS-CoV2 infection, the region where the microbiome was sampled (gut or oral/upper respiratory tract), and experimental variables (contained within study). The model was significant (p-value < 0.001). Explanatory power <0.01 is not shown, and significant fractions are indicated with *** (p-value < 0.001).
